# Volume distribution of nodal domains of random band-limited functions

**DOI:** 10.1007/s00440-017-0813-x

**Published:** 2017-11-03

**Authors:** Dmitry Beliaev, Igor Wigman

**Affiliations:** 1 Mathematical Institute, University of Oxford, Andrew Wiles Building, Radcliffe Observatory Quarter, Woodstock Road, OX2 6GG Oxford, UK; 2 Department of Mathematics, King’s College London, Strand, London, WC2R 2LS UK

**Keywords:** 58J50, 60F99, 35P20

## Abstract

We study the volume distribution of nodal domains of families of naturally arising Gaussian random fields on generic manifolds, namely random band-limited functions. It is found that in the high energy limit a typical instance obeys a deterministic universal law, independent of the manifold. Some of the basic qualitative properties of this law, such as its support, monotonicity and continuity of the cumulative probability function, are established.

## Introduction

A conjecture of Berry [3] was the original motivation for the study of nodal lines of a random plane wave. Nowadays, the study of the nodal lines of random plane waves and other smooth Gaussian fields is a well developed research area of its own right, also having numerous connections to other areas of mathematics and mathematical physics. We refer to a survey by Nazarov and Sodin [10] for more information. Bogomolny and Schmit [4] argued that some properties of the nodal domains of random plane wave behave similarly to the analogous properties of the critical percolation clusters corresponding to the square lattice; these include their number and the distribution of their areas.

It turns out that a lot of techniques could be extended to more general ensembles of random functions that we will discuss later on. In this paper we discuss the distribution of nodal domains’ areas and length of their boundaries; we are following the footsteps of Nazarov and Sodin [9, 11], who developed some novel techniques to study the total number of nodal domains of smooth fields, and Sarnak and Wigman [12] who extended their tools to study finer questions of counting nodal domains of a given topological type and their mutual positions (“nestings”). Some of the methods in our paper are similar to these of the aforementioned papers; in particular, we borrow several technical results from these manuscripts. Some marked differences from these will be discussed at the end of the introduction.

Let $(\mathcal {M},g)$ be a compact smooth Riemannian *n*-manifold. For a smooth function $f:\mathcal {M}\rightarrow \mathbb {R}$ the nodal domains are the connected components of the complement $\mathcal {M}{\setminus } f^{-1}(0)$ of the nodal set, and we denote $\varOmega (f)$ to be the collection of all nodal domains of *f*, and for $t>0$ we denote $\mathcal {N}(f;t)$ to be the number of nodal domains $\omega \in \varOmega (f)$ of volume

$$\begin{aligned} {\text {Vol}}(\omega )<t, \end{aligned}$$

where ${\text {Vol}}={\text {Vol}}_n$ is the *n*-dimensional volume on $\mathcal {M}$. We also define

$$\begin{aligned} \mathcal {N}(f) = \mathcal {N}(f;\infty ) = |\varOmega (f)| \end{aligned}$$

to be the total number of nodal domains of *f*. In this paper we will investigate the behaviour of *N*(*f*; *t*) for several classes of random functions *f*.

Before introducing the most general result we would like to discuss one particular case (whose scaling limit is Berry’s random monochromatic waves) which is easy to explain and is representative in the proof of the general result. It is well-known that the space of spherical harmonics of degree *l* is of dimension $2l+1$; let $\{\phi _{l,i}\}_{i=1\ldots 2l+1}$ be an arbitrary $L^2$-orthonormal basis. Define a random Gaussian spherical harmonic

1.1 $$\begin{aligned} f_l=\sqrt{\frac{1}{2l+1}}\sum \limits _{i=1}^{2l+1} c_i \phi _{l,i}, \end{aligned}$$

where $c_i$ are i.i.d. standard Gaussian variables. The normalizing constant is chosen so that

$$\begin{aligned} \mathbb {E}[|f(z)|^2]=1 \end{aligned}$$

for every $$z\in \mathcal {S}^{2}$$.

For the total number of nodal domains of $f_{l}$ Nazarov and Sodin [9, 11] proved that there exists a constant $c_{0}>0$ (“Universal Nazarov–Sodin Constant”) so that

1.2 $$\begin{aligned} \mathbb {E}\left[ \left| \frac{\mathcal {N}(f_{l})}{l^{2}} - 4\pi c_{0} \right| \right] \rightarrow 0, \end{aligned}$$

i.e. that $\frac{\mathcal {N}(f_{l})}{l^{2}}$ converges to the constant $4\pi \cdot c_{0}>0$ *in mean*, where $4\pi ={\text {Vol}}_{2}(\mathcal {S}^{2})$ is the surface area of the unit 2-sphere. The constant $c_{0}$ in (1.2) will be denoted $c_{0}=c(2,1)$ as a particular case of a more general situation below [see (1.8)]. Our first principal result refines this.

### Theorem 1

Let $f_l$ be the random spherical harmonic of degree *l*. Then the following holds:

1. There exists a monotone non-decreasing function
  $$\begin{aligned} \varPsi =\varPsi _{2,1}:(0,\infty )\rightarrow \mathbb {R}_{+ }, \end{aligned}$$
  so that for all continuity points *t* of $\varPsi$ we have
  1.3 $$\begin{aligned} \mathbb {E}\left[ \left| \frac{\mathcal {N}(f_{l};t/l^{2})}{\mathcal {N}(f_{l})} - \varPsi (t) \right| \right] \rightarrow 0,\qquad l\rightarrow \infty . \end{aligned}$$
  (Notation $\varPsi _{2,1}$ will be clear from the formulation of Theorem 2.)
2. Let
  $$\begin{aligned} t_0=\pi j_{0,1}^2=18.168\ldots \end{aligned}$$
  where $$j_{0,1}\approx 2.4048$$ is the first zero of the Bessel function $$J_0$$. Then the function $$\varPsi $$ defined above vanishes on $$[0,t_{0})$$, and is strictly increasing for $$t>t_{0}$$.

As a monotone increasing function $\varPsi$ is continuous outside a countable set of jumps $\mathcal {T}_{0}=\mathcal {T}_{0;2;1} = \{t_{k}\}_{k=1}^{\infty }$; hence (1.3) holds for all $t\in \mathbb {R}_{>0}{\setminus }\mathcal {T}_{0}$. The question whether we should expect for some genuine “distinguished” numbers $t\in \mathcal {T}_{0}$ that accumulate a positive proportion of nodal domain areas with strictly positive probability turned out to be quite tricky. On one hand, it is a priori possible to construct examples of spherical harmonics admitting lots of nodal domains of similar volume $\approx t$, for some candidate $t>0$ for $\mathcal {T}_{0}$. However, we may show that this situation is unstable w.r.t. small perturbations of the given spherical harmonics, by evaluating the derivatives of the volumes of these nodal domains w.r.t. the perturbation, and proving most of them to be bounded away from 0, with high probability.

The latter procedure seems very difficult to implement in order to rigorously rule out atoms, since the typical situation is more complicated, so that, even though the nodal domains of volume $\approx t$ before perturbation have been perturbed sufficiently, there might occur *other* domains, whose volume will be $\approx t$ *after* the perturbation. Nevertheless the above heuristic could serve as an argument in favour of believing in no “distinguished” areas, accumulating a positive proportions of nodal domain areas, at all, as in the following conjecture (or at least $|\mathcal {T}_{0}|<\infty$ is finite). This is also our grounds for believing the analogous conjecture in the more general setting (see Conjecture 2 below); if our intuition is correct, then $\varPsi$ is also differentiable everywhere, both in the restricted case $\varPsi =\varPsi _{2,1}$ (see the second part of Conjecture 1), and in the more general scenario.

### Conjecture 1

Let $\varPsi$ be the function prescribed by Theorem 1.

1. The set of jumps of $\varPsi$ is empty, i.e. $\mathcal {T}_{0} = \emptyset$, and $\varPsi$ is continuous.
2. The function $\varPsi$ is everywhere differentiable on $(t_0,\infty )$ and its derivative (the probability density of the limiting distribution of nodal areas) is strictly positive.

Let us make a few remarks on Theorem 1. The spherical harmonics have a natural scale 1 / *l*, hence the natural area scaling is $t/l^2$; the area of a typical nodal domain is of order of magnitude $\frac{1}{l^2}$. Since the spherical harmonics are eigenfunctions of the spherical Laplacian, there is deterministic a lower bound on the nodal domains area. This will follow from the Faber–Krahn inequality which, in the 2-dimensional case, states that for a domain of area *A* its first Dirichlet eigenvalue of the Laplacian is at least $\pi j_{0,1}^2/A$. This implies that $\varPsi (t)=0$ for $t<t_0$. One may formulate and prove similar results on the distribution of lengths of nodal domains boundaries (equivalently, connected components of the nodal set $f_{l}^{-1}(0)$), called the *nodal components* of $f_{l}$ (see also Sect. 6).

All the other principal results of this paper are in the same spirit as Theorem 1 but in different, less specialized, settings. The two main settings are: Euclidian (or “scale invariant”) case, and band-limited ensembles on manifolds; one recovers the former as scaling limits of the band-limited ensembles around every point of the given manifold. Below we briefly describe the various settings of random ensembles of functions treated in this paper.

### Euclidian random fields

Here we are interested in centred Gaussian functions $F:\mathbb {R}^n\rightarrow \mathbb {R}$; it is a well known fact (Kolmogorov’s Theorem) that the distribution of a centred Gaussian field is completely determined by its covariance kernel

$$\begin{aligned} K(x,y)=\mathbb {E}[F(x)\cdot F(y)]. \end{aligned}$$

We will be interested in *isotropic* fields i.e. the fields such that

$$\begin{aligned} K(x,y)=K(|x-y|), \end{aligned}$$

which means that *F* is invariant under translations and rotations. From now on we assume that all our fields on $\mathbb {R}^n$ are isotropic, and moreover, we will also assume that *F* is normalized so that $K(0)=\mathbb {E}[F^2(x)] =1.$

It is known that the such covariance kernel *K* can be expressed as the Fourier transform of a measure $\rho$, called the *spectral measure*. In many cases it is more convenient to describe the field *F* in term of its spectral measure instead of the covariance kernel. Given the spectral measure, there is an alternative way of constructing the random function *F*. It can be constructed as the Gaussian vector in the Hilbert space $\mathcal {H}$ which is Fourier image of the symmetric space $L^2_{\mathrm {sym}}(\rho )$. In particular this means that if $\{\phi _k\}_{k}$ is an orthonormal basis in $\mathcal {H}$, then

1.4 $$\begin{aligned} F=\sum \zeta _k \phi _k, \end{aligned}$$

where $\zeta _k$ are i.i.d Gaussian random variables. This series a.s. diverges in $\mathcal {H}$ but, under suitable assumptions on $\rho$, converges a.s., pointwise or in some other sense, to a well-defined function. The nature of the convergence depends on the properties of $\rho$; in cases of our most interest the series (1.4) converges locally in $C^k$ for every *k*. One of the most important motivational examples is the random plane wave:

#### Definition 1

The *random plane wave* or the *monochromatic wave* with energy $E=k^2$ is the centred Gaussian field on $\mathbb {R}^{2}$ with the covariance kernel

$$\begin{aligned} K(x,y)=J_0(k|x-y|), \end{aligned}$$

where $J_0$ is the zeroth Bessel function.

Since the spectral measure is supported on the unit circle, the random plane wave is a solution of Helmholz equation

1.5 $$\begin{aligned} \varDelta f+k^2 f=0 \end{aligned}$$

on $\mathbb {R}^{2}$. One may think of the random plane wave as a “random” solution of (1.5). It is possible to express the random plane wave in terms of an orthonormal basis in the corresponding Hilbert space via the formula (1.4). A canonical basis for this Hilbert space is given in terms of Bessel functions $J_k$, and, in polar coordinates $x=re^{i\theta }$, the function *F* could be written as

1.6 $$\begin{aligned} F(re^{i\theta })=\mathfrak {R}\sum _{n=-\infty }^\infty c_n J_{|n|}( kr)e^{i n \theta }, \end{aligned}$$

where the coefficients $c_n$ are i.i.d. standard complex Gaussians. More details about the construction of the random plane wave and its relation to the random spherical harmonics could be found in [9].

A direct computation shows that the covariance kernel of this function is indeed $K(x,y)=J_0(k|x-y|)$. The spectral measure of *F* is the normalized Lebesgue measure on the circle of radius *k*. Since plane waves with different values of *k* differ by the scaling, it is natural to fix $k=1$. The description in terms of the spectral measure has a natural generalization to the higher dimensions:

#### Definition 2

The random plane wave in $\mathbb {R}^n$ is the Gaussian field whose spectral measure is the normalized $(n-1)$-dimensional Lebesgue measure on $S^{n-1}\subset \mathbb {R}^n$.

In Theorem 5 below we will show that under relatively mild conditions on the spectral measure, an analogue of Theorem 1 holds for *F*. Theorem 5 will be proved in great generality, though the most important, relevant for Theorem 1, is the case of the random monochromatic plane-wave, of significant importance by itself.

### Band-limited functions

Let $(\mathcal {M},g)$ be a compact Riemanian *n*-manifold, then the eigenfunctions of the Laplacian $\{\phi _i\}_{i\ge 1}$ form an orthonormal basis of $L^{2}(\mathcal {M})$. We denote the square roots of eigenvalues by $0=t_0\le t_1\le t_2 \ldots$ i.e. satisfying

$$\begin{aligned} \varDelta \phi _i+t_i^2\phi _i=0. \end{aligned}$$

For a fixed $\alpha \in [0,1)$ and $T\gg 0$ a large spectral parameter we define the $\alpha$-band-limited functions (corresponding to *T*)

1.7 $$\begin{aligned} f(x) =f_{\alpha ;T}(x)= \sum \limits _{\alpha T \le t_{j} \le T} c_{j}\phi _{j}(x). \end{aligned}$$

where $c_{j}$ are independent real Gaussian variables of mean 0 and variance 1. For $\alpha =1$ we define $f_{1;T}$ by

$$\begin{aligned} f(x) =f_{1;T}(x)= \sum \limits _{T-\eta (T) \le t_{j} \le T} c_{j}\phi _{j}(x), \end{aligned}$$

where $\eta$ is a function growing to infinity slower than *T*, i.e. $\eta (T)\rightarrow \infty$ as $T\rightarrow \infty$ and $\eta (T)=o(T)$.

The random spherical harmonic (1.1) defined above is an $\alpha =1$ band-limited function on the unit sphere $\mathcal {S}^{2}$ with $\alpha =1$ and $\eta (T)=O(T^{1/2})$. For the total number of nodal domains of the band limited functions Nazarov and Sodin [11, 13] proved that for every $\alpha \in [0,1]$, $n\ge 2$ there exist a constant $c(n,\alpha )>0$ (“Nazarov–Sodin constant”^1^) satisfying

1.8 $$\begin{aligned} \mathbb {E}\left[ \left| \frac{\mathcal {N}_{\varOmega }(f)}{T^{n}} - c(n,\alpha ) \cdot {\text {Vol}}_{n}(\mathcal {M}) \right| \right] \rightarrow 0. \end{aligned}$$

Sarnak and Wigman [12] refined the latter result (1.8) for counting the number of nodal domains (or components) of *f* of a given topological class; they also found an elegant way to formulate it in terms of convergence of random probability measures consolidating all topological types into a universal deterministic probability measure that conserves all the topologies. Gayet and Welshinger [8] proved lower (and upper) bounds for the expected number of domains of a given topological class in a different ensemble of random polynomials in the high degree limit. We will show below that a result similar to Theorem 1 holds for band-limited functions.

#### Theorem 2

Let $f=f_{\alpha ;T}$ be a band-limited function on some *n*-dimensional compact manifold $\mathcal {M}$. There exists a monotone non-decreasing function

$$\begin{aligned} \varPsi =\varPsi _{n;\alpha }:(0,\infty )\rightarrow \mathbb {R}_{+ }, \end{aligned}$$

so that for all continuity points *t* of $\varPsi$ we have

1.9 $$\begin{aligned} \mathbb {E}\left[ \left| \frac{\mathcal {N}_{\varOmega }(f;t/T^{n/2})}{\mathcal {N}_{\varOmega }(f)} - \varPsi (t) \right| \right] \rightarrow 0, \qquad T\rightarrow \infty . \end{aligned}$$

Importantly, the function $\varPsi$ prescribed by Theorem 2 depends on dimension *n*, but not on the manifold.

### Some properties of the limiting distribution $\varPsi _{n;\alpha }$

Here we investigate the most basic property of $\varPsi _{n;\alpha }$, i.e. its monotonicity. We need to distinguish between $\alpha <1$, where the corresponding distribution function $\varPsi _{n;\alpha }$ is strictly positive and increasing everywhere (Theorem 3), and $\alpha =1$, where the behaviour of the function $\varPsi _{n;1}$ is more complicated (Theorem 4).

#### Theorem 3

For every $n\ge 2$, $\alpha < 1$ the function $\varPsi _{n;\alpha }$ as above is strictly increasing on $\mathbb {R}_{+}$.

#### Theorem 4

For every $n\ge 2$ the function $\varPsi _{n;1}$ vanishes on $[0,t_{0}]$ where

$$\begin{aligned} t_{0}=t_{0}(n)=\frac{\pi ^{n/2}}{\varGamma (n/2+1)}j^n_{n/2-1,1} \end{aligned}$$

is the volume of the ball of radius $j_{n/2-1,1}$ – the first zero of the Bessel function $J_{n/2-1}$. Moreover, $\varPsi _{n;1}$ is strictly increasing on $(t_0,\infty )$.

Motivated by similar arguments to Conjecture 1 (see the couple of paragraphs preceding Conjecture 1) it is only natural to conjecture the following:

#### Conjecture 2

The function $\varPsi _{n;\alpha }$ is continuous, everywhere differentiable. For $\alpha <1$ the derivative $\frac{d\varPsi _{n;\alpha }(t)}{dt}>0$ is everywhere positive, whereas for $\alpha =1$ the same holds for $t>t_{0}$.

### Main ideas and the plan of the paper

Our general strategy is similar to [11, 12]; for a start, in Sect. 2.1 we give the necessary background on the behaviour of band-limited functions in the semiclassical limit. Roughly speaking, we show that on small scale bigger than 1 / *T* the rescaled version $f_{\alpha ;T}$ of the band-limited function is well-approximated locally around every point $x\in \mathcal {M}$ by its limit $\mathfrak {g}_{n,\alpha }$ defined on the tangent space $T_{x}\mathcal {M}$. Importantly, $\mathfrak {g}_{n,\alpha }$ is universal, i.e. it does not depend on manifold or *x*, and it is sufficiently explicit for us to conduct local computations. This will allow us to prove the main results for $\mathfrak {g}_{n,\alpha }$, and then use the effective convergence to deduce the results for the band-limited functions. In Sect. 2.2 we formulate several Kac–Rice type results that will yield universal upper bounds on various local quantities like the number of nodal domains or nodal components.

In Sect. 3 we discuss the behaviour of Gaussian fields on $\mathbb {R}^n$. We formulate and prove Theorem 5, a Euclidean version of Theorem 2, holding under very mild conditions on a random field, including all of the $\mathfrak {g}_{n,\alpha }$. The proof of Theorem 2 is based on *a priori* upper bounds via Kac–Rice, and an ergodic theorem, yielding the existence of the scaling limit of the volume distribution. Towards the end of Sect. 3 where Theorems 6 and 7 on the distribution function $\varPsi _{n,\alpha }$ are proved; these are Euclidean analogues of Theorems 3 and 4, covering the generic case $\alpha <1$, and $\alpha =1$ respectively. The proofs of Theorems 6 and 7 are rather similar. Here our first goal is establishing the existence of a *deterministic* function with nodal domain containing the origin of volume approximating the given number. Then we show that the same holds for functions approximating the postulated one in $C^1$-norm (see Lemma 10); finally we claim that the latter happens with positive probability. The translation invariance of the underlying random fields yields that, as the claimed result holds near origin with positive probability, it holds with positive *density*.

In Sect. 4 we prove that since functions $f_{\alpha ;T}$ and $\mathfrak {g}_{n,\alpha }$ are close to each other (after coupling and rescaling), their respective numbers of nodal domains of restricted volume are close with high probability (this is quantified in Proposition 1). In Sect. 5 we prove the main theorems of the paper. The proofs are based on *semi-locality* of nodal domains, that is, that most of the nodal domains of $f_{\alpha ; T}$ are neither too small nor too long, i.e. that the semi-local approximation by $\mathfrak {g}_{n,\alpha }$ captures most of the nodal domains of $f_{\alpha ;T}$. This allows to infer the results on $f_{\alpha ;T}$ from the analogous results on $\mathfrak {g}_{n,\alpha }$. Finally, in Sect. 6 we make some final remarks about the proof, in particular we explain that with some minor modification our methods imply similar results for the distribution of the surface volume of the nodal components or even for the joint distribution of the volumes of nodal domains and boundary volumes.

Despite the fact that our general approach follows the footsteps of Nazarov and Sodin [11, 13] and Sarnak and Wigman [12] (and Canzani and Sarnak [7] for the full support statement in the monochromatic case $\alpha =1$), our case offers new significant challenges on both the Euclidean stage and the inference of the Riemannian one, and also for proving the full support statement on the volume distribution. First, as we are interested in the number of nodal domains of a given approximate volume, we need to refine the techniques to control the volumes of the perturbed nodal domains rather than their mere number; though it shares some similarities with [12], the continuous variable *t* makes the analysis more challenging as compared to the purely discrete (and hence atomic) case in [12]. While the more refined technical work is a relatively standard application of techniques in differential geometry (Lemma 10), if some $t>0$ happens to be an atom of the limiting distribution, it does not guarantee that *t* maintains its mass after the perturbation. In fact, as a result of the perturbation its mass might spread in an infinitesimal neighbourhood $(t-\epsilon ,t+\epsilon )$ of *t*; this is the reason why the main results are only formulated for continuity points of the limit distribution function $\varPsi$.

Second, as part of the full support statement for the limit distribution (Theorems 3 and 4), we need to construct a deterministic function in some space of functions with a nodal domain of a prescribed volume. In the monochromatic case this is only possible if it obeys the Faber–Krahn inequality; in this case we show that the converse is also valid, whence are are building on [7] and refine it for our needs. Finally, while an analogue of the ergodic approach of Nazarov and Sodin [11, 13] yields the mere *existence* of $\varPsi (t)$ for every given $t>0$ the properties of $\varPsi$ are far from being obvious. In fact, it could be that the atoms of the limit distribution corresponding to $\varPsi$ concentrate all the probability.

## Necessary background

### Semiclassical properties of band-limited functions and their scaling limits

In this section we introduce a few facts about band-limited functions. The results are stated without proofs, for more detailed discussion we refer the readers to [12, Section 2.1] and references therein.

For the band-limited function $f_{\alpha ;T}$ we have the covariance function

$$\begin{aligned} K_{\alpha ; T}(x,y):= \mathbb {E}\left[ f_{\alpha ;T}(x)\cdot f_{\alpha ;T}(y)\right] =\sum \limits _{\alpha T \le t_{j} \le T} \phi _{j}(x)\phi _{j}(y), \end{aligned}$$

with the same conventions as above for $\alpha =1$. The following semiclassical approximation, independent of $\mathcal {M}$, holds (see [12, Section 2.1] with case $\alpha =1$ due to Canzani and Hanin [5, 6]):

2.1 $$\begin{aligned} \widetilde{K_{\alpha }} (T;x,y) := \frac{1}{D_{\alpha }(T)}K_{\alpha } (T;x,y) = B_{n,\alpha }(T\cdot d(x,y)) + O\left( T^{-1}\right) , \end{aligned}$$

uniformly for $x,y\in \mathcal {M}$, where *d*(*x*, *y*) is the (geodesic) distance in $\mathcal {M}$ between *x* and *y*,

$$\begin{aligned} D_{\alpha }(T)=\frac{1}{{\text {Vol}}(\mathcal {M})} \int \limits _{\mathcal {M}}K_{\alpha }(T;x,x)d{\text {Vol}}(x), \end{aligned}$$

and for $$w\in \mathbb {R}^{n}$$

2.2 $$\begin{aligned} B_{n,\alpha }(w) = B_{n,\alpha }(|w|) = \frac{1}{|A_{\alpha }|} \int \limits _{A_{\alpha }}e^{2\pi i\langle w,\xi \rangle } d\xi \end{aligned}$$

where $A_{\alpha }=\left\{ w:\, \alpha \le |w|\le 1 \right\}$ and in the case $\alpha =1$ the *n*-dimensional measure $d\xi$ is replaced by $(n-1)$-dimensional surface measure on the unit sphere.

We may differentiate both sides of (2.1) to obtain asymptotic expression for *finitely* many derivatives of $K_{\alpha }$. By appropriately normalizing $f_{\alpha }$ we may assume w.l.o.g that $\widetilde{K_{\alpha }}$ is the covariance of $f_{\alpha }$ and we will neglect this difference from this point on.

Around each point $x\in \mathcal {M}$ we define the scaled random fields $f_{x;T}$ (we drop $\alpha$ from notations) on a big ball (after scaling) lying on the Euclidian tangent space $\mathbb {R}^{n}\cong T_{x}\mathcal {M}$ with the use of the exponential map and an isometry $I_{x}:\mathbb {R}^{n}\rightarrow T_{x}\mathcal {M}$, $\varPhi _{x}=\exp _{x}\circ I_{x}:\mathbb {R}^{n}\rightarrow \mathcal {M}$ via

2.3 $$\begin{aligned} f_{x;T}(u) := f_{T}(\varPhi _{x}(u/T)), \end{aligned}$$

with the covariance function

$$\begin{aligned} K_{x;T}(u,v) := \mathbb {E}[f_{x;T}(u)\cdot f_{x;T}(v)] = K_{T}(\varPhi _{x}(u/T),\varPhi _{x}(v/T)). \end{aligned}$$

Observe that locally $\varPhi _{x}$ is almost an isometry: for each $\xi$ there exists $r_{0}=r_{0}(\xi )$ such that if $\mathcal {D}\subseteq B_{x}(r_{0})$ is a smooth domain in $\mathcal {M}$, then

2.4 $$\begin{aligned} |{\text {Vol}}_{\mathcal {M}} (\mathcal {D}) -{\text {Vol}}_{\mathbb {R}^n}(\varPhi ^{-1}_{x}(\mathcal {D}))|< \xi , \end{aligned}$$

uniformly w.r.t. $x\in \mathcal {M}$ ($r_{0}$ is assumed to be sufficiently small so that the exponential map is $1-1$). By the scaling (2.3) we obviously have

$$\begin{aligned} \mathcal {N}\left( f,\frac{t}{T^{n}};x,\frac{R}{T}\right) \approx \mathcal {N}\left( f_{x;T},t;x,R\right) , \end{aligned}$$

the precise meaning will be given in (4.3). This means that studying $f_{\alpha ;T}$ and $f_{x;T}$ is essentially equivalent. Finally, from (2.1) we see that the covariance kernel of $f_{x,T}$ converges to

$$\begin{aligned} r_{n,\alpha }(u,v)=B_{n,\alpha }(|u-v|). \end{aligned}$$

This suggests to define the local scaling limit $\mathfrak {g}_{n,\alpha }$ to be a Gaussian function in $\mathbb {R}^n$ with this covariance kernel. Alternatively, it could be defined by its spectral measure which by (2.2) is the normalized Lebesgue measure on $A_\alpha$ (or the normalized surface area on $A_1$ for $\alpha =1$). It is important to point out that the scaling limit is universal: it does not depend on *x* or $\mathcal {M}$, but only on *n* and $\alpha$.

From the above it follows that for *T* sufficiently large, the covariance kernels of $f_{x,T}$ are asymptotic to those of $\mathfrak {g}_{n,\alpha }$. However it is not obvious that this implies that with high probability $f_{x,T}$ and $\mathfrak {g}_{n,\alpha }$ are close (which is essential for our approach). One obstacle we need to overcome is that the random fields $f_{x,T}$ and $\mathfrak {g}_{n,\alpha }$ are defined on different probability spaces. However, it is still possible to *couple* $f_{x,T}$ and $\mathfrak {g}_{n,\alpha }$ so that $f_{x,T}-\mathfrak {g}_{n,\alpha }$ is small in $C^{1}$ on a ball of arbitrarily big radius. To establish the latter statement (in a suitable sense to be formulated rigorously) one would restrict the respective fields on a sufficiently dense grid and use the approximation of the multivariate distribution to approximate the finite-dimensional Gaussian variables. One then extends the respective fields to be defined smoothly w.r.t. the continuous variable on the relevant domain, still maintaining the $C^{1}$-approximation property. The above procedure was carried out rigorously in [13, Lemma 4].

#### Lemma 1

([13, Lemma 4]) Given $R>0$ and $b>0$, there exists $T_{0}=T_{0}(R,b)$ such that for all $T\ge T_{0}$ we have

$$\begin{aligned} \mathbb {E}\left[ \Vert f_{x;T}-\mathfrak {g}_{n,\alpha }\Vert _{C^{1}(\overline{B}(2R))}\right] < b. \end{aligned}$$

### The Kac–Rice premise

In this section we collect a number of *local* results required below. The Kac–Rice formula is a powerful tool for computing moments of local quantities, and in principle it is capable of expressing *any* moment of a local quantity of a given random Gaussian field *F* in terms of explicit, albeit complicated, Gaussian expectations, or, equivalently, in terms of the covariance kernel.

Let $m\le n$, $F:\mathcal {D}\rightarrow \mathbb {R}^{m}$ be a smooth random field on a domain $\mathcal {D}\subseteq \mathbb {R}^{n}$, and $\mathcal {Z}(F;\overline{\mathcal {D}})$ be either the $(n-m)$-volume of $F^{-1}(0)$ (for $m<n$), or the number of the discrete zeros (for $m=n$). For example, if $H:\mathcal {M}\rightarrow \mathbb {R}$ is a random field and $F=\nabla H|_{\mathcal {D}}:\mathcal {M}\rightarrow \mathbb {R}^{n}$ is its gradient restricted to a coordinate patch, then $\mathcal {Z}(F,\overline{\mathcal {D}})$ counts the number of critical points of *H* on $\mathcal {D}$. We set $J_{F}(x)$ to be the (random) Jacobi matrix of *F* at *x*, and define the “zero density” of *F* at $x\in \mathcal {D}$ as the conditional Gaussian expectation

2.5 $$\begin{aligned} K_{1}(x) = K_{1;F}(x)=\phi _{F(x)}(0)\cdot \mathbb {E}[|\det J_{F}(x)| \big | F(x)=0], \end{aligned}$$

where $\phi _{F(x)}(0,\ldots ,0)$ is the *m*-variate Gaussian density of *F*(*x*) evaluated at $(0,\ldots , 0)\in \mathbb {R}^{m}$; if the distribution of *F*(*x*) in non-degenerate with covariance matrix $D=D(x)$, then we have

$$\begin{aligned} \phi _{F(x)}(0,\ldots ,0) = \frac{1}{(2\pi )^{m/2}\sqrt{\det {D}}}. \end{aligned}$$

With the above notation the Kac–Rice formula (meta-theorem) states that, under some non-degeneracy condition on *F*,

$$\begin{aligned} \mathbb {E}[\mathcal {Z}(F;\mathcal {D})]=\int \limits _{\mathcal {D}}K_{1}(x)dx. \end{aligned}$$

Concerning the sufficient conditions that guarantee that (2.5) holds, a variety of results is known [1, 2]. The following version of Kac–Rice merely requires the non-degeneracy of the values of *F* (vs. the non-degeneracy of $(F,J_{F}(x))$ in the appropriate sense, as in some more classical sources), to our best knowledge, the mildest sufficient condition. All random fields considered in this paper satisfy these assumptions.

#### Lemma 2

(Standard Kac–Rice [2, Theorem 6.2]) Let $F:\mathcal {D}\rightarrow \mathbb {R}^{m}$ be an a.s. $C^{2}$-smooth Gaussian field, such that for every $x\in \mathcal {D}$ the distribution of the random vector $F(x)\in \mathbb {R}^{m}$ is non-degenerate Gaussian, with zero almost surely not a critical value. Then

2.6 $$\begin{aligned} \mathbb {E}[\mathcal {Z}(F;\mathcal {D})]=\int \limits _{\mathcal {D}}K_{1}(x)dx \end{aligned}$$

with the zero density $K_{1}(x)$ as in (2.5).

Condition (iv) of [2, Theorem 6.2] that zero is not a critical level is automatically satisfied by all $C^{2}$-smooth Gaussian fields; this is known as Bulinskaya’s Lemma, see [2, Proposition 6.12].

The following lemma is an upper bound for either the number of critical points of a random field or its restriction to a hypersphere as a result of an application of Kac–Rice on coordinate patches.

#### Lemma 3

([12, Corollary 2.3]) Let $\mathcal {D}\subseteq \mathbb {R}^{m}$ be a domain and $F:\mathcal {D}\rightarrow \mathbb {R}$ an a.s. $C^{2}$-smooth stationary Gaussian random field, such that for $x\in \mathcal {D}$ the distribution of $\nabla F(x)$ is non-degenerate Gaussian.

1. For $r>0$ let $\mathcal {A}(F;r)$ be the number of critical points of *F* inside $B(r)\subseteq \mathcal {D}$. Then
  $$\begin{aligned} \mathbb {E}[\mathcal {A}(F;r)] = O({\text {Vol}}(B(r))), \end{aligned}$$
  where the constant involved in the ‘*O*’-notation depends on the law of *F* only.
2. For $r>0$ let $\widetilde{\mathcal {A}}(F;r)$ be the number of critical points of the restriction $F|_{\partial B(r)}$ of *F* to the sphere $\partial B(r)\subseteq \mathcal {D}$. Then
  $$\begin{aligned} \mathbb {E}[\widetilde{\mathcal {A}}(F;r)]= O({\text {Vol}}(\partial B(r))), \end{aligned}$$
  where the constant involved in the ‘*O*’-notation depends on the law of *F* only.

The following estimate is the upper bound part of the (precise) Kac–Rice formula applied to the band limited functions.

#### Lemma 4

([13, Lemma 2] and [12, Lemma 7.8]) For $x\in \mathcal {M}$, $r>0$ let $\mathcal {N}_{\varOmega }(f_{\alpha ;T};x,r)$ be the number of nodal domains of $f_{\alpha ;T}$ entirely lying in $B(x,r)\subseteq \mathcal {M}$: the geodesic ball of radius *r* centred at *x*. Then

$$\begin{aligned} \mathbb {E}[\mathcal {N}_{\varOmega }(f_{\alpha ;T};x,r)]= O(r^{n}\cdot T^{n}), \end{aligned}$$

with constant involved in the $`O'$-notation depending on $\mathcal {M}$ and $\alpha$ only.

## Distribution of nodal domain areas for Euclidian fields

### Notation and statement of the main result on Euclidian fields

#### Notation and basic setup

Let *f* be a smooth function, $t>0$, and $R>0$. We denote $\mathcal {N}(f,t;R)$ to be the number of domains $\omega \in \varOmega (f)$ of *f* lying entirely in *B*(*R*) of volume

$$\begin{aligned} {\text {Vol}}_{n}(\omega )\le t; \end{aligned}$$

note that

$$\begin{aligned} \mathcal {N}(f;R)=\mathcal {N}(f,\infty ;R) \end{aligned}$$

is the total number of nodal domains lying inside *B*(*R*), as considered by Nazarov and Sodin [9].

We are interested in the asymptotic distribution^2^ of the nodal domain volumes, that is, the asymptotic behaviour of $\mathcal {N}(F,t;R)$ as $R\rightarrow \infty$, $t>0$ fixed; throughout this section we will tacitly assume that *F* is stationary, so that its spectral measure makes sense. We would like to establish the limit

3.1 $$\begin{aligned} \varPsi _{\rho }(t) := \lim \limits _{R\rightarrow \infty }\frac{\mathcal {N}(F,t;R)}{\mathcal {N}(F;R)} \end{aligned}$$

in mean, and, moreover, that $\varPsi _{\rho }(t)$ is a distribution function, i.e.

$$\begin{aligned} \lim \limits _{t\rightarrow \infty }\varPsi _{\rho }(t)=1. \end{aligned}$$

The latter will follow once having established the former (in the proper sense) via the obvious *deterministic* upper bound

$$\begin{aligned} \mathcal {N}(F;R)- \mathcal {N}(F,t;R) \le \frac{{\text {Vol}}B(R)}{t} \end{aligned}$$

for the number of domains of volume greater than *t*.

Following Nazarov and Sodin [11] we assume that the spectral measure $\rho$ of *F* satisfies the following axioms:

$$(\rho 1)$$
The measure $\rho$ has no atoms (if and only if the action of the translations is ergodic by Grenander–Fomin–Maruyama, see [13, Theorem 3]).
$$(\rho 2)$$
For some $$p>4$$,
  $$\begin{aligned} \int \limits _{\mathbb {R}^{n}}|\lambda |^{p}d\rho (\lambda ) < \infty \end{aligned}$$
  (this ensures the a.s. $C^{2}$-smoothness of *F*).
$$(\rho 3)$$
The spectral support ${\text {supp}}\rho$ does not lie in a linear hyperplane. (This ensures that the random Gaussian field, together with its gradient is not degenerate.)

For this model Nazarov and Sodin [11] proved that there exists a constant $c(\rho )\ge 0$ (the “Nazarov–Sodin constant”) so that

3.2 $$\begin{aligned} \frac{\mathcal {N}(F;R)}{{\text {Vol}}B(R)} \rightarrow c(\rho ) \end{aligned}$$

both in mean and a.s.

It is shown in [11] that under the additional mild condition the constant $c(\rho )$ is strictly positive. We do not want to discuss these technicalities, so instead we will use the assumption

$$(\rho 4)$$
The Nazarov–Sodin constant $c(\rho )$ is positive.

Sometimes we will invoke a stronger axiom:

$$(\rho 4^{*})$$
The support of the spectral measure $\rho$ has non-empty interior.

#### Existence of limiting distribution $\varPsi _{\rho }$

##### Theorem 5

Let $F:\mathbb {R}^{n}\rightarrow \mathbb {R}$ be a stationary random field whose spectral measure $\rho$ satisfies the axioms $(\rho 1)-(\rho 3)$, then

$$\begin{aligned} \frac{\mathcal {N}(F,t;R)}{ {\text {Vol}}(B(R))} \end{aligned}$$

converges in mean as $R\rightarrow \infty$. If we additionally assume the axiom $(\rho 4)$, then the limit

$$\begin{aligned} \lim \limits _{R\rightarrow \infty }\frac{\mathcal {N}(F;R)}{ {\text {Vol}}(B(R))} = c(\rho )>0 \end{aligned}$$

does not vanish, so that we can define the normalized limit

3.3 $$\begin{aligned} \varPsi _{\rho }(t):=\lim \limits _{R\rightarrow \infty }\frac{\mathcal {N}(F,t;R)}{c(\rho )\cdot {\text {Vol}}(B(R))}. \end{aligned}$$

Since the total number of nodal domains of *F* lying inside *B*(*R*) was proven to be asymptotic to

$$\begin{aligned} \mathcal {N}(F;R)\sim c(\rho )\cdot {\text {Vol}}B(R), \end{aligned}$$

[see (3.2)], (3.3) may be equivalently read as

$$\begin{aligned} \frac{\mathcal {N}(F,t;R)}{\mathcal {N}(F;R)}\rightarrow \varPsi _{\rho }(t), \end{aligned}$$

[cf. (3.1)]; this limit could be proven in mean, see the proof of Theorem 2 in Sect. 5.1. Theorem 5 in particular implies that for every $t>0$ the expected number $\mathcal {N}(F,t;R)$ of nodal domains of *F* of volume at most *t* lying in *B*(*R*) is

3.4 $$\begin{aligned} \mathbb {E}[\mathcal {N}(F,t;R)]= c(\rho )\cdot \varPsi _{\rho }(t)\cdot {\text {Vol}}(B(R))(1+o_{R\rightarrow \infty }(1)), \end{aligned}$$

with concentration: for every $\epsilon >0$

3.5 $$\begin{aligned} \lim \limits _{R\rightarrow \infty } \mathcal {P}\left\{ \left| \frac{\mathcal {N}(F,t;R)}{{\text {Vol}}B(R)} - c(\rho )\cdot \varPsi _{\rho }(t)\right| > \epsilon \right\} =0, \end{aligned}$$

via Chebyshev’s inequality.

#### Some properties of $\varPsi _{\rho }(t)$

##### Theorem 6

(Lower bound on domains in the generic case) Assume that the spectral measure of *F* satisfies axioms $(\rho 1)-(\rho 3)$ and $(\rho 4^{*})$. Then $\varPsi _{\rho }(0)= 0$, and $\varPsi _{\rho }(\cdot )$ is strictly increasing on $[0,\infty )$; in particular, for every $t>0$ we have $\varPsi _{\rho }(t)> 0$.

For the random plane wave (RPW) the situation is slightly different. Its spectral measure does not satisfy ($\rho 4^{*}$) but satisfies ($\rho 4$). The corresponding function $\varPsi =\varPsi _{RPW}$ vanishes up to a certain explicit threshold. The precise statement is given in the following theorem.

##### Theorem 7

Let $F=F_{RPW}$ be the random plane wave in $\mathbb {R}^n$. As its spectral measure satisfies axioms $(\rho 1)-(\rho 4)$, the function $\varPsi _{RPW}=\varPsi _{\rho }$ defined as in Theorem 5 exists. Define

$$\begin{aligned} t_{0}=t_{0}(n)=\frac{\pi ^{n/2}}{\varGamma (n/2+1)}j^n_{n/2-1,1} \end{aligned}$$

as in Theorem 4. Then the following holds:

1. For every nodal domain $\omega$ of $F_{RPW}$ we have ${\text {Vol}}(\omega )\ge t_0,$ and hence, in particular, we have
  $$\begin{aligned} \varPsi _{RPW}(t) =0, \qquad t<t_{0} \end{aligned}$$
2. Moreover, for $n=2$ the function $\varPsi$ is strictly increasing on $[t_0,\infty )$

The remaining part of Sect. 3 is dedicated to the proofs of Theorems 5, 6 and 7.

### Integral-Geometric Sandwich

Let $\varGamma \subseteq \mathbb {R}^{n}$ be a hypersurface (a curve for $n=2$). For $u\in \mathbb {R}^{n}$, $r>0$ and $t>0$ we denote $\mathcal {N}(\varGamma ,t;u,r)$ the number of domains of $\varGamma$ of *n*-dimensional volume bounded by *t* lying entirely in the radius-*r* ball $B_{u}(r)$ centred at *u*. Similarly, define $\mathcal {N}^{*}(\varGamma ,t;u,r)$ by relaxing the condition to domains merely *intersecting* $\overline{B}_{u}(r)$. We use the shortcuts

$$\begin{aligned} \mathcal {N}(g,t;u,r) :=\mathcal {N}(g^{-1}(0),t;u,r), \end{aligned}$$

respectively

$$\begin{aligned} \mathcal {N}^{*}(g,t;u,r) :=\mathcal {N}^{*}(g^{-1}(0),t;u,r), \end{aligned}$$

and $\mathcal {N}(\cdot ,\cdot ;r) :=\mathcal {N}(\cdot ,\cdot ;0,r)$ (resp. $\mathcal {N}^{*}(\cdot ,\cdot ;r) :=\mathcal {N}^{*}(\cdot ,\cdot ;0,r)$), consistent to Sect. 3.1.1. Finally, let $\mathcal {N}(g;u,r)=\mathcal {N}(g,\infty ;u,r)$ be the total number of domains lying inside $B_{0}(r)$.

#### Lemma 5

(cf. [13, Lemma 1]) Let $\varGamma$ be a closed hypersurface. Then for $0<r<R$, $t>0$,

3.6 $$\begin{aligned} \begin{aligned} \frac{1}{{\text {Vol}}(B(r))}\int \limits _{B(R-r)} \mathcal {N}(\varGamma ,t;u,r)du&\le \mathcal {N}(\varGamma ,t;R) \\&\le \frac{1}{{\text {Vol}}(B(r))}\int \limits _{B(R+r)} \mathcal {N}^{*}(\varGamma ,t;u,r)du. \end{aligned} \end{aligned}$$

#### Proof

We follow along the lines of the proof of [13, Lemma 1]: For a connected component $\gamma$ of $\varGamma$ denote $\mathcal {A}(\gamma )$ to be the area of the domain having $\gamma$ as its outer boundary.

Let $\gamma$ be any connected component of $\varGamma$. Define

$$\begin{aligned} G_{*}(\gamma ) = \bigcap \limits _{v\in \gamma } B_{v}(r) = \{u:\gamma \subseteq B_{u}(r) \} \end{aligned}$$

and

$$\begin{aligned} G^{*}(\gamma ) = \bigcup \limits _{v\in \gamma } \overline{B_{v}(r)} = \{u:\gamma \cap \overline{B_{u}(r)}\ne \emptyset \}. \end{aligned}$$

We have for every $v\in \gamma$,

$$\begin{aligned} G_{*}(\gamma ) \subseteq B_{v}(r) \subseteq G^{*}(\gamma ), \end{aligned}$$

and thus, in particular,

3.7 $$\begin{aligned} {\text {Vol}}(G_{*}(\gamma )) \le {\text {Vol}}(B_{v}(r)) \le {\text {Vol}}(G^{*}(\gamma )). \end{aligned}$$

Summing up (3.7) for all connected components $\gamma \subseteq \varGamma$ lying inside *B*(*R*), corresponding to domains of volume at most *t*, we obtain

3.8 $$\begin{aligned} \begin{aligned} \sum \limits _{\begin{array}{c} \gamma :\, \mathcal {A}(\gamma )\le t\\ \gamma \subseteq B(R) \end{array}}{\text {Vol}}(G_{*}(\gamma ))&\le {\text {Vol}}(B(r))\cdot \mathcal {N}(\varGamma ,t;R) \le \sum \limits _{\begin{array}{c} \gamma :\, \mathcal {A}(\gamma )\le t\\ \gamma \subseteq B(R) \end{array}} {\text {Vol}}(G^{*}(\gamma )) . \end{aligned} \end{aligned}$$

Writing the volume as an integral

$$\begin{aligned} {\text {Vol}}(G_{*}(\gamma )) = \int \limits _{G_{*}(\gamma )}du, \end{aligned}$$

and exchanging the order of summation and integration we obtain

3.9 $$\begin{aligned} \sum \limits _{\begin{array}{c} \gamma :\, \mathcal {A}(\gamma )\le t \\ \gamma \subseteq B(R) \end{array}}{\text {Vol}}(G_{*}(\gamma )) \ge \int \limits _{B(R-r)}\left[ \sum \limits _{\begin{array}{c} \gamma :\, \mathcal {A}(\gamma )\le t\\ \gamma \subseteq B_{u}(r) \end{array}} 1 \right] du = \int \limits _{B(R-r)} \mathcal {N}(\varGamma ,t;u,r) du, \end{aligned}$$

since if $u\in B(R-r)$ then $B_{u}(r)\subseteq B(R)$. Similarly,

3.10 $$\begin{aligned} \sum \limits _{\begin{array}{c} \gamma :\, \mathcal {A}(\gamma )\le t\\ \gamma \subseteq B(R) \end{array}} {\text {Vol}}(G^{*}(\gamma )) \le \int \limits _{B(R+r)}\left[ \sum \limits _{\begin{array}{c} \gamma :\, \mathcal {A}(\gamma )\le t\\ \gamma \cap \overline{B_{u}(r)}\ne \emptyset \end{array}}1 \right] du = \int \limits _{B(R+r)}\mathcal {N}^{*}(\varGamma ,t;u,r)du, \end{aligned}$$

since if $\gamma \subseteq B(R)$ and for some *u*, $\overline{B_{u}(r)}\cap \gamma \ne \emptyset$, then necessarily $u\in B(R+r)$. The statement of the present lemma then follows from substituting (3.9) and (3.10) into (3.8), and dividing both sides by ${\text {Vol}}B(r)$. $\square$

### Proof of Theorem 5

#### Proof

Let $t>0$ be given, and fix $r>0$; we apply (3.6) to $\varGamma =F^{-1}(0)$:

$$\begin{aligned} \begin{aligned}&\left( 1-\frac{r}{R}\right) ^{n}\frac{1}{{\text {Vol}}B(R-r)}\int \limits _{B(R-r)} \frac{\mathcal {N}(F,t;u,r)}{{\text {Vol}}(B(r))}du \le \frac{\mathcal {N}(F,t;R)}{{\text {Vol}}(B(R))}\\&\quad \le \left( 1 + \frac{r}{R}\right) ^{n}\cdot \frac{1}{{\text {Vol}}B(R+r)} \int \limits _{B(R+r)} \frac{\mathcal {N}^{*} (F,t;u,r)}{{\text {Vol}}(B(r))}du\\&\quad \le \left( 1 + \frac{r}{R}\right) ^{n}\frac{1}{{\text {Vol}}B(R+r)}\int \limits _{B(R+r)} \frac{\mathcal {N}(F,t;u,r)+\mathcal {C}(u,r;t,F)}{{\text {Vol}}(B(r))}du, \end{aligned} \end{aligned}$$

where $\mathcal {C}(u,r;t,F)$ is the number of domains $\omega \in \varOmega (F)$ intersecting $\partial B_{u}(r)$, of volume

$$\begin{aligned} {\text {Vol}}(\omega ) \le t, \end{aligned}$$

bounded by the total number of critical points of the restriction $F|_{\partial B_{u}(r)}$ of *F* to the hypersphere $\partial B_{u}(r)$, and

$$\begin{aligned} {\text {Vol}}(B(R\pm r)) = {\text {Vol}}(B(R))\cdot \left( 1\pm \frac{r}{R} \right) ^{n}. \end{aligned}$$

We rewrite $\mathcal {N}(F,t;u,r) = \mathcal {N}(\tau _{u} F,t;r)$, where $\tau _{u}$ is the translation operator

$$\begin{aligned} (\tau _{u}F)(x) = F(u+x). \end{aligned}$$

Choose *r* so small that $(1\pm r/R)^n$ are $\epsilon$-close to 1, then we have

3.11 $$\begin{aligned}&\left( 1-\epsilon \right) \frac{1}{{\text {Vol}}B(R-r)}\int \limits _{B(R-r)} \frac{\mathcal {N}(\tau _{u}F,t;r)}{{\text {Vol}}(B(r))}du \le \frac{\mathcal {N}(F,t;r)}{{\text {Vol}}(B(R))} \nonumber \\&\qquad \le \left( 1 + \epsilon \right) \frac{1}{{\text {Vol}}B(R+r)}\int \limits _{B(R+r)} \frac{\mathcal {N}(\tau _{u}F,t;r)+ \mathcal {C}(\tau _{u} F,t;r)}{{\text {Vol}}(B(r))}du. \end{aligned}$$

Note that for every *r*, *t* the functional

$$\begin{aligned} F\mapsto \varUpsilon _{r;t}(F):=\frac{\mathcal {N}(F,t;r)}{{\text {Vol}}(B(r))} \end{aligned}$$

(and its translations) is measurable, and since the number of nodal domains is bounded by the number of critical points, this functional has finite expectation by Lemma 3, part (1). It then follows from the ergodic theory that both

$$\begin{aligned}\frac{1}{{\text {Vol}}B(R+r)}\int \limits _{B(R+r)} \frac{\mathcal {N}_{\cdot } (\tau _{u}F,t;r)}{{\text {Vol}}(B(r))}du\end{aligned}$$

and

$$\begin{aligned}\frac{1}{{\text {Vol}}B(R-r)}\int \limits _{B(R-r)} \frac{\mathcal {N}_{\cdot }(\tau _{u}F,t;r)}{{\text {Vol}}(B(r))}du\end{aligned}$$

converge to (the same) limit in $L^1$

$$\begin{aligned} \frac{1}{{\text {Vol}}B(R)}\int \limits _{B(R)} \frac{\mathcal {N}_{\cdot }(\tau _{u}F,t;r)}{{\text {Vol}}(B(r))}du \rightarrow c_{r;t}(\rho ):=\mathbb {E}[\varUpsilon _{r;t}]. \end{aligned}$$

(Initially only the existence of the limit is known; it equals the mean value by $L^{1}$-convergence.)

Observe that, if we get rid of

$$\begin{aligned} \mathcal {C}(\tau _{u} F,t;r) \end{aligned}$$

on the rhs of (3.11) then, up to $\pm \epsilon$, both the lhs and the rhs of (3.11) would converge to the same limit $c_{r;t}(\rho )$ (in either $L^{1}$ or a.s.). We will be able to get rid of $\mathcal {C}(\tau _{u} F,t;r)$ asymptotically for *r* large; it will yield that as $r\rightarrow \infty$, we have the limit $c_{r;t}(\rho )\rightarrow c_{t}(\rho )$ satisfying

3.12 $$\begin{aligned} \frac{\mathcal {N}(F,t;R)}{{\text {Vol}}(B(R))} \rightarrow c_{t}(\rho ). \end{aligned}$$

Setting

$$\begin{aligned}\varPsi _{\rho }(t) :=\frac{c_{t}(\rho )}{c(\rho )}\end{aligned}$$

with $c(\rho )$ the Nazarov–Sodin constant (3.2) will ensure that $\varPsi _{\rho }(t)$ obeys (3.3) of Theorem 5. That $\varPsi _{\rho }$ is a distribution function will then follow easily:

$$\begin{aligned}t\mapsto c_{t}(\rho )\end{aligned}$$

is monotone nondecreasing by (3.12); $\varPsi _{\rho }(\infty )=1$ since $c_{\infty }(\rho ) = c(\rho )$ is the Nazarov–Sodin constant.

To see that indeed we can get rid of $\mathcal {C}(\tau _{u} F,t;r)$ we use the same ergodic argument as before, now applied to the map

$$\begin{aligned} F\mapsto \frac{\mathcal {C}(F,t;r)}{{\text {Vol}}(B(r))}, \end{aligned}$$

yielding the $L^{1}$ limit

$$\begin{aligned} \frac{1}{{\text {Vol}}B(R+r)}\int \limits _{B(R+r)}\frac{\mathcal {C}(\tau _{u} F,t;r)}{{\text {Vol}}(B(r))}\rightarrow a_{r;t}(\rho ), \end{aligned}$$

whence

$$\begin{aligned} a_{r;t}(\rho ) = \mathbb {E}\left[ \frac{\mathcal {C}(\tau _{u} F,t;r)}{{\text {Vol}}(B(r))}\right] = O\left( \frac{1}{r}\right) \end{aligned}$$

by the second part of Lemma 3. Hence (3.11) implies

$$\begin{aligned} \mathbb {E}\left[ \left| \frac{\mathcal {N}(F,t;R)}{{\text {Vol}}(B(R))} - c_{r;t}(\rho )\right| \right] = O\left( \epsilon +\frac{1}{r}\right) ; \end{aligned}$$

this proves the existence of the limits

$$\begin{aligned} c_{t}(\rho ):=\lim \limits _{r\rightarrow \infty }c_{r;t}(\rho ), \end{aligned}$$

and, as it was mentioned above,

$$\begin{aligned}\varPsi _{\rho }(t) :=\frac{c_{t}(\rho )}{c(\rho )},\end{aligned}$$

is the distribution function satisfying the $L^{1}$-convergence (3.3) we were after in Theorem 5. $\square$

#### Remark 1

This proof also shows a.s. convergence but we will not need that for the rest (i.e. the Riemannian case).

### Proofs of Theorems 6 and 7

#### Proof of Theorem 6

Since the spectral measure satisfies conditions $(\rho 1)-(\rho 3)$, the existence of the limiting distribution $\varPsi$ is guaranteed by Theorem 5. We only have to show that $\varPsi$ is strictly increasing.

The proof of Theorem 6 will consist of three steps. First we will construct a deterministic function with nodal domain of given area. After that we will show that the probability that there is a random function with almost the same nodal domain is positive. Finally we show that this implies that the expected density of such nodal domains is positive.

The first step is essentially the same as the argument in [12, Proposition 5.2]. By condition $(\rho 4^{*})$ the interior of the support of $\rho$ is non-empty. Let us assume that $B(\zeta _0,r_0)\subset {\text {supp}}\rho$. We are going to show that this implies that for every compact *Q* the functions from $\mathcal {H}(\rho )$ are dense in $C^k(Q)$ for every *k*.

Let $\phi _n$ be a sequence of positive $C^\infty$ functions with supports converging to $\zeta _0$ which approximate $\delta$-function at $\zeta _0$. Functions $\psi _n(\zeta )=\phi _n(\zeta )+\phi _n(-\zeta )$ and all their derivatives belong to $L^2_{\mathrm {sym}}(\rho )$ and hence their Fourier transforms belong to $\mathcal {H}$. The Fourier transform of $\psi _n$ converges to $e^{2\pi i <\zeta _0,x>}$ in any $C^k(Q)$ and its derivatives $\partial ^\alpha \psi _n$ converge to $(-2\pi x)^\alpha e^{2\pi i <\zeta _0,x>}$, where $x^\alpha =\prod x_i^{\alpha _i}$. This proves that we can approximate any polynomial (times a fixed plane wave). Since the polynomials are dense in $C^k(Q)$, we have that $\mathcal {H}$ is dense in $C^k(Q)$.

In particular, for given $t>0$ and $\epsilon >0$ we can take a $C^1$ function such that its nodal domain around the origin is inside some disc *B*(0, *r*) and has area *t*. Moreover, we can assume that its gradient is bounded away from zero in some neighbourhood of the boundary of this nodal domain. By approximating this function by a function from $\mathcal {H}$ and applying Lemma 10 we obtain a function $f_t$ from $\mathcal {H}$ such that its nodal domain around the origin has area $\epsilon$-close to *t* and its gradient is bounded away from zero on the nodal line. $\square$

Without loss of generality we may assume that $\mathcal {H}$ norm of $f_t$ is 1 and extend it to an orthonormal basis $\phi _i$. In this basis *F* could be written as

$$\begin{aligned} F=c_0 f_t +\phi , \end{aligned}$$

where $c_0$ is a standard Gaussian random variable and $\phi$ is a Gaussian function independent of $c_{0}$ (spanned by all additional basis functions $\phi _i$). Since $f_t$ is non-degenerate, the probability that

$$\begin{aligned} \max \{c_{0}|f|,c_{0}|\nabla f_t|\}>1 \end{aligned}$$

in some (fixed) neighbourhood of the nodal domain containing the origin is strictly positive. Let *r* be such that the nodal domain of $f_t$ is in *B*(0, *r*), then if $C^1(B(0,2r))$ norm of $\phi$ is sufficiently small (see Lemma 10 for the precise statement), than the volume of the nodal domain of *F* is $\epsilon$-close to that of $f_t$. It is a standard fact (cf. [9]) that the probability that $\phi$ have small norm is strictly positive. This completes the proof that the area of the nodal domain containing the origin is $2\epsilon$ close to *t* is strictly positive.

We can pack $\mathbb {R}^2$ with infinitely many copies of the disc of radius 2*r* such that this covering is locally uniformly finite. By translation invariance, for each disc the probability that there is a nodal domain of area $2\epsilon$-close to *t* is strictly positive (independently of the disc). This implies that

$$\begin{aligned} \frac{\mathcal {N}(F,t+2\epsilon ;R)-\mathcal {N}(F,t-2\epsilon ;R)}{{\text {Vol}}(B(R))}>c >0 \end{aligned}$$

for some positive *c* and all sufficiently large *R*. This prove that $\varPsi (t+2\epsilon )-\varPsi (t-2\epsilon )>c$ which means that $\varPsi$ is strictly increasing for $t>t_0$. $\square$

#### Proof of Theorem 7

The proof of this theorem is almost exactly the same as the proof of Theorem 6, but the first deterministic step is different. The main difference is that the spectral measure is supported on the sphere, hence its support has empty interior and the space $\mathcal {H}$ is not dense in $C^k(Q)$.

The fact that $\varPsi$ vanishes for $t<t_0$ is completely deterministic and follows from the Faber–Krahn inequality. Recall that the random plane wave is a solution to $\varDelta f+f=0$. Let $\omega$ be a nodal component of a plane wave *F*. Since *F* has a constant sign in $\omega$ it must be the first eigenfunction of Laplacian in $\omega$. This implies that the first eigenvalue in $\omega$ is 1. By the Faber–Krahn inequality the volume of $\omega$ is greater than the volume of the ball for which the main eigenvalue is also equal to 1. It is a well known fact that $|x|^{1-n/2}J_{n/2-1}(|x|)$ is an eigenfunction of the Laplacian in $\mathbb {R}^n$ with eigenvalue 1. Its nodal domain containing the origin is a ball of radius $j_{n/2-1,1}$ and area $t_0$. This proves that the volume of every nodal domain is bounded below by $t_0$.

To prove the last part of the theorem we first claim that for every $t\ge t_0$ there is a domain $\varOmega _t$ such that ${\text {Vol}}(\varOmega _t)=t$ and the main eigenvalue of the Laplacian is 1. We define $\varOmega _L$ to be the union of the cylinder of length *L* and radius $j_{n/2-1,1}$ with two hemispherical caps at both ends. For $L=0$ this is the ball of the radius $j_{n/2-1,1}$ and as $L\rightarrow \infty$ it converges to the infinite cylinder. Since $\varOmega _L$ depends on *L* continuously, the main eigenvalue $\lambda _L$ changes continuously from $\lambda _0=1$ to $\lambda _\infty =(j_{(n-1)/2-1,1}/j_{n/2-1,1})^2>0$. By rescaling $\varOmega _L$ by $\sqrt{\lambda _L}$ we obtain a family of domains such that their main eigenvalue is 1 and their volumes change continuously from $t_0$ to infinity. Let us relabel this family and say that $\varOmega _t$ is the element of the family with volume *t*.

After that we follow the strategy from [7]. By Whitney approximation theorem we can construct a domain with analytic boundary which is an arbitrary small perturbation of $\varOmega _t$. By continuity of the main eigenvalue we can assume that it is arbitrary close to 1. By rescaling the domain we obtain a domain with analytic boundary, main eigenvalue 1 and volume $\epsilon$ close to *t*. Abusing notation we call this domain $\varOmega _t$ as well.

Let $g_t>0$ be the main eigenfunction of Laplacian in $\varOmega _t$. Since the boundary is analytic, it could be extended to a small neighbourhood *B* of $\partial \varOmega _t$ in such a way that $\inf \Vert \nabla g_t\Vert >0$ in a small tubular neighbourhood of $\partial \varOmega _t$. By Lax-Malgrange Theorem for every $b>0$ there is a function $f_t$ which is a *global* solution to (1.5) such that $\Vert f_t-g_t\Vert _{C^1(B)}<b$. If *b* is sufficiently small, then by Lemma 10 there is a nodal domain of $f_t$ with volume $\epsilon$ close to ${\text {Vol}}(\varOmega _t)$. This proves that there is a *deterministic* non-degenerate plane wave with a nodal domain such that its volume is $2\epsilon$-close to *t*. The rest of the proof is exactly the same as in Theorem 6. $\square$

## Local results

### Local setting and statement of the local result

Recall that $\mathfrak {g}_{n,\alpha }$ is the “scaling limit” of $f_{n,\alpha ;T}$ as $T\rightarrow \infty$, around every point $x\in \mathcal {M}$ in the following sense (cf. Sect. 2.1). We know that for *T* large, their covariance functions $K_{\alpha ,T}$ and $r_{n,\alpha }$ are, after scaling, asymptotic to each other, uniformly on a large ball $B(R)\subseteq \mathbb {R}^{n}$. By Lemma 1 they could be coupled so that

$$\begin{aligned} \Vert f_{x;T}-\mathfrak {g}\Vert \end{aligned}$$

is arbitrarily small in $C^{1}$-norm on *B*(*R*) (or *B*(2*R*)).

For $u\in \mathcal {M}$, $r>0$ sufficiently small, $t>0$ and $g:\mathcal {M}\rightarrow \mathbb {R}$ a smooth (deterministic) function denote $\mathcal {N}(g,t;u,r)$ (resp. $\mathcal {N}^{*}(g,t;u,r)$) the number of domains $\omega \in \varOmega (g)$ of $g^{-1}(0)$ of volume

$$\begin{aligned} {\text {Vol}}(\omega )\le t \end{aligned}$$

lying entirely inside (resp. intersecting) the geodesic ball $B_{u}(r)\subseteq \mathcal {M}$. Theorem 8, to follow immediately, states that, with the coupling as above, unless *t* is a discontinuity point of the limiting volume distribution (i.e. atoms of the $\varPsi _{\rho }$ obtained from an application of Theorem 5 on $\mathfrak {g}_{n,\alpha }$), after appropriate rescaling, the nodal domain volumes of $f_{\alpha ;T}$ are *locally* point-wise approximated by the ones of $\mathfrak {g}_{n,\alpha }$. Otherwise, if $t_{0}$ is an atom of the said $\varPsi _{\rho }$ (its existence is unlikely, by Conjecture 2), then the corresponding probability mass may spread in an interval $(t_{0}-\epsilon ,t_{0}+\epsilon )$ ($\epsilon >0$ arbitrarily small) beyond our control (see the proof of Proposition 1 below).

#### Theorem 8

(cf. [13, Theorem 5]) Let $f(x)=f_{\alpha ;T}(x)$ be the random band limited functions (1.7), $\varPsi =\varPsi _{n,\alpha }$ the distribution function prescribed by Theorem 5 applied on $\mathfrak {g}_{n,\alpha }$. Then for every continuity point $t>0$ of $\varPsi _{n,\alpha }(\cdot )$, $x\in \mathcal {M}$ and $\epsilon >0$ we have

4.1 $$\begin{aligned} \lim \limits _{R\rightarrow \infty }\limsup \limits _{T\rightarrow \infty } \mathcal {P}\left\{ \left| \frac{\mathcal {N}\left( f,\frac{t}{T^{n}};x,\frac{R}{T}\right) }{c(n,\alpha )\cdot {\text {Vol}}(B(R))}-\varPsi (t)\right| > \epsilon \right\} = 0, \end{aligned}$$

where $c(n,\alpha )>0$ is the Nazarov–Sodin constant of $\mathfrak {g}_{n,\alpha }$.

The rest of this section is dedicated to giving a proof of Theorem 8.

### Proof of Theorem 8

We formulate the following proposition that will imply Theorem 8. The proof is postponed till Sect. 4.4 after some preparatory work in Sect. 4.3.

#### Proposition 1

Let $x\in \mathcal {M}$, $R>0$, small numbers $\delta ,\xi ,\eta >0$ be given, and $t>0$. Then, for $R>0$ sufficiently big, outside of an event of probability at most $\delta$, for all $T>T_{0}(R,\delta ,\xi )$ we have

4.2 $$\begin{aligned} \begin{aligned} \mathcal {N}(\mathfrak {g}_{n,\alpha },t-\xi ;R-1) - \eta \cdot R^{n}&\le \mathcal {N}\left( f_{x;T},t;R\right) \\&\le \mathcal {N}(\mathfrak {g}_{n,\alpha },t+\xi ;R+1) + \eta \cdot R^{n}. \end{aligned} \end{aligned}$$

#### Proof of Theorem 8 assuming Proposition 1

Let $x\in \mathcal {M}$, $\epsilon >0$ be fixed, and $\xi ,\eta >0$ be given small numbers. We observe that by the definition (2.3) of the scaled fields $f_{x;T}$ and (2.4), for *T* sufficiently big (depending on *R*) we have

4.3 $$\begin{aligned} \mathcal {N}\left( f_{x;T},t-\xi ;R\right) \le \mathcal {N}\left( f,\frac{t}{T^{n}};x,\frac{R}{T}\right) \le \mathcal {N}\left( f_{x;T},t+\xi ;R\right) \end{aligned}$$

Hence Proposition 1 together with (4.3) holding for *T* sufficiently big (depending on *R*) imply that for *T* sufficiently big, outside an event of arbitrarily small probability,

$$\begin{aligned} \begin{aligned} \mathcal {N}(\mathfrak {g}_{n,\alpha },t-2\xi ;R-1)-\eta \cdot R^{n}&\le \mathcal {N}\left( f,\frac{t}{T^{n}};x,\frac{R}{T}\right) \\&\le \mathcal {N}(\mathfrak {g}_{n,\alpha },t+2\xi ;R+1) + \eta \cdot R^{n}. \end{aligned} \end{aligned}$$

Choose

4.4 $$\begin{aligned} \eta <\frac{c(n,\alpha )\cdot \epsilon \cdot {\text {Vol}}B(1)}{2} \end{aligned}$$

so that

4.5 $$\begin{aligned}&\mathcal {P}\left\{ \left| \frac{\mathcal {N}\left( f,\frac{t}{T^{n}};x,\frac{R}{T}\right) }{c(n,\alpha )\cdot {\text {Vol}}B(R)} - \varPsi (t)\right|>\epsilon \right\} \nonumber \\&\le \mathcal {P}\left\{ \left| \frac{\mathcal {N}(\mathfrak {g}_{n,\alpha },t+2\xi ;R+1)}{c(n,\alpha )\cdot {\text {Vol}}B(R)} - \varPsi (t) \right|> \epsilon - \frac{\eta }{c(n,\alpha )\cdot {\text {Vol}}B(1)} \right\} \nonumber \\&\quad + \mathcal {P}\left\{ \left| \frac{\mathcal {N}(\mathfrak {g}_{n,\alpha },t-2\xi ;R-1)}{c(n,\alpha )\cdot {\text {Vol}}B(R)} - \varPsi (t) \right| > \epsilon -\frac{\eta }{c(n,\alpha ) {\text {Vol}}B(1)} \right\} . \end{aligned}$$

Now, bearing in mind our choice (4.4), we have that $\epsilon -\frac{\eta }{c(n,\alpha ) \cdot {\text {Vol}}B(1)} > \frac{\epsilon }{2},$ so that

$$\begin{aligned} \begin{aligned}&\left\{ \left| \frac{\mathcal {N}(\mathfrak {g}_{n,\alpha },t+2\xi ;R+1)}{c(n,\alpha )\cdot {\text {Vol}}B(R)} - \varPsi (t) \right|> \epsilon -\frac{\eta }{c(n,\alpha )\cdot {\text {Vol}}B(1)} \right\} \subseteq \\&\left\{ \left| \frac{\mathcal {N}(\mathfrak {g}_{n,\alpha },t+2\xi ;R+1)}{c(n,\alpha )\cdot {\text {Vol}}B(R)} - \varPsi (t+2\xi ) \right| > \frac{\epsilon }{2} - (\varPsi (t+2\xi )-\varPsi (t)) \right\} , \end{aligned} \end{aligned}$$

and thus

4.6 $$\begin{aligned} \begin{aligned}&\mathcal {P}\left\{ \left| \frac{\mathcal {N}(\mathfrak {g}_{n,\alpha },t+2\xi ;R+1)}{c(n,\alpha )\cdot {\text {Vol}}B(R)} - \varPsi (t)\right|> \epsilon -\frac{\eta }{c(n,\alpha )\cdot {\text {Vol}}B(1)} \right\} \\&\le \mathcal {P}\left\{ \left| \frac{\mathcal {N}(\mathfrak {g}_{n,\alpha },t+2\xi ;R+1)}{{\text {Vol}}B(R)} - \varPsi (t+2\xi ) \right| > \frac{\epsilon }{2} - (\varPsi (t+2\xi )-\varPsi (t)) \right\} \rightarrow 0 \end{aligned} \end{aligned}$$

as $R\rightarrow \infty$ by Theorem 5, and the continuity of $\varPsi (\cdot )$ at *t*. Similarly, as $R\rightarrow \infty$,

4.7 $$\begin{aligned} \mathcal {P}\left\{ \left| \frac{\mathcal {N}(\mathfrak {g}_{n,\alpha },t-2\xi ;R-1)}{c(n,\alpha ){\text {Vol}}B(R)} - \varPsi (t) \right| > \epsilon -\frac{\eta }{c(n,\alpha )\cdot {\text {Vol}}B(1)} \right\} \rightarrow 0. \end{aligned}$$

The above proves that for each $R>0$ sufficiently large there exists $T_{0}=T_{0}(R)$, such that (4.5) holds for $T>T_{0}$; the latter could be made arbitrarily small by (4.6) and (4.7). This is precisely the statement of Theorem 8. $\square$

### Some preparatory results towards the proof of Proposition 1

In course of the proof of Proposition 1 we will need to exclude some exceptional events. Let $\delta >0$ be a small parameter that will control the probabilities of the discarded events, $b,\beta >0$ be small parameters that will control the quality of the various approximations, $\eta >0$ will control the number of discarded domains, and $M,Q>0$ large parameters. Given *R* and *T* big we define

$$\begin{aligned} \varDelta _{1}= & {} \varDelta _{1}(R,T;b)= \{ \Vert f_{x;T}-\mathfrak {g}_{n,\alpha }\Vert _{C^{1}(\overline{B}(2R))} \ge b\},\\ \varDelta _{2}= & {} \varDelta _{2}(R,T;M) = \left\{ \Vert f_{x,T}\Vert _{C^{2}(\overline{B}(2R))} \ge M \right\} ,\\ \varDelta _{3}= & {} \varDelta _{3}(R;M) = \left\{ \Vert \mathfrak {g}_{n,\alpha }\Vert _{C^{2}(\overline{B}(2R))} \ge M \right\} \end{aligned}$$

and the “unstable event”

$$\begin{aligned} \varDelta _{4}(R;\beta ) = \left\{ \min \limits _{u\in \overline{B}(2R)}\max \{|\mathfrak {g}_{n,\alpha }(u)|, |\nabla \mathfrak {g}_{n,\alpha }(u)|\} \le 2\beta \right\} . \end{aligned}$$

Moreover let $\varDelta _{5}$ be the event

$$\begin{aligned} \begin{aligned}&\varDelta _{5}(R;\eta ,Q) \\&=\left\{ \left| \left\{ \text {components } \omega \in \varOmega (\mathfrak {g}_{n,\alpha }):\,\omega \subseteq B(2R),\, {\text {Vol}}_{n-1}(\partial \omega )>Q \right\} \right| >\eta R^{n}\right\} \end{aligned} \end{aligned}$$

that there is a significant number of nodal domains of $\mathfrak {g}_{n,\alpha }$ entirely lying in *B*(2*R*) whose boundary volume is at least *Q*. The boundary of such a domain is comprised of a number of nodal components; below we will argue that, with high probability, each of the boundary components is of bounded volume and their total number is bounded (see the proof of Lemma 9).

The following is a simple corollary of Lemma 1.

#### Lemma 6

For a given sufficiently big $R>0$ and small $b,\delta >0$ there exists $T_{0}=T_{0}(R,b,\delta )$ so that for $T>T_{0}$ the probability of $\varDelta _{1}$

4.8 $$\begin{aligned} \mathcal {P}(\varDelta _{1}(R,T;b))<\delta . \end{aligned}$$

The following lemma yields a bound on the probabilities $\mathcal {P}(\varDelta _{2})$ and $\mathcal {P}(\varDelta _{3})$.

#### Lemma 7

1. For every $R,\delta >0$ there exists $M=M(R,\delta )>0$ so that
  4.9 $$\begin{aligned} \mathcal {P}(\varDelta _{3}(R;M)) < \delta . \end{aligned}$$
2. For $R,\delta >0$ there exist $M(R,\delta )>0$ and $T_{0}=T_{0}(R)$ so that
  4.10 $$\begin{aligned} \mathcal {P}(\varDelta _{2}(R,T;M))< \delta \end{aligned}$$
  for all $$T>T_{0}$$.

#### Proof

For (4.9) we may choose *M* to be

$$\begin{aligned} M=\delta ^{-1}\mathbb {E}[\Vert \mathfrak {g}_{n,\alpha }\Vert _{C^{2}(\overline{B}(2R))} ], \end{aligned}$$

and the latter expectation is finite by [1, Theorem 2.1.1]. The estimate (4.9) with this value of *M* follows from Chebyshev’s inequality.

In order to establish (4.10) we observe that by “Sudakov-Fernique comparison inequality” [1, Theorem 2.2.3] and (2.1) applied to both $K_{x,T}$ and its derivatives, for all $M_1$ such that

$$\begin{aligned} \mathbb {E}[\Vert \mathfrak {g}_{n,\alpha }\Vert _{C^{2}(\overline{B}(2R))} ] <M_{1} \end{aligned}$$

there exists $T_{0}=T_{0}(R,M_{1})$ such that for all $T>T_{0}$

$$\begin{aligned} \mathbb {E}\left[ \Vert f_{x,T}\Vert _{C^{2}(\overline{B}(2R))}\right] < M_{1}. \end{aligned}$$

Hence we can set $M=\delta ^{-1}M_{1}$ and (4.10) will follow from Chebyshev’s inequality as before.

The event $\varDelta _{4}$ means that the nodal set of $\mathfrak {g}_{n,\alpha }$ is relatively unstable. For small $\beta$ the probability of this event can be made arbitrary small. This is Lemma 7 from [11]:

#### Lemma 8

(cf. [11]) For $R>0$ and $\delta >0$ there exist $\beta >0$ such that

4.11 $$\begin{aligned} \mathcal {P}\left( \varDelta _{4}(R,\beta ) \right) < \delta . \end{aligned}$$

Finally, for $\varDelta _{5}$ we have the following bound:

#### Lemma 9

For every $\eta >0$ and $\delta >0$ there exist $Q>0$ and $R_{0}=R_{0}(\eta ,\delta$) such that for all $R>R_{0}$ the probability of $\varDelta _{5}$ is

4.12 $$\begin{aligned} \mathcal {P}\left( \varDelta _{5}(R;\eta ,Q)\right) < \delta . \end{aligned}$$

#### Proof

In what follows we argue that most of the domains have a bounded number of components lying in the boundary, and with high probability, each has bounded $n-1$-volume. First, using Nazarov–Sodin’s (3.2), there exists a number *A* sufficiently big so that the probability that the total number of nodal domains of $\mathfrak {g}_{n,\alpha }$ entirely lying inside *B*(2*R*) is bigger than $A\cdot R^{n}$ is

4.13 $$\begin{aligned} \mathcal {P}(\varDelta _{6}(A)):=\mathcal {P}\left\{ \mathcal {N}(\mathfrak {g}_{n,\alpha };2R) > A\cdot R^{n} \right\} <\delta /2, \end{aligned}$$

so we may exclude this unlikely event $\varDelta _{6}=\varDelta _{6}(A)$.

Now we are going to show that the number of boundary components of most of the nodal domains of $\mathfrak {g}_{n,\alpha }$ lying in *B*(2*R*) is bounded; outside of $\varDelta _{6}$ there are less than $A\cdot R^{n}$. To this end we use the *nesting graph* introduced in [12]. Let $G=(V,E)$ be the graph with the set of vertexes *V* being the collection of all nodal domains

$$\begin{aligned}V=\{\omega \in \varOmega (\mathfrak {g}_{n,\alpha }):\, \omega \subseteq B(2R)\}\end{aligned}$$

of $\mathfrak {g}_{n,\alpha }$ lying entirely in *B*(2*R*), and an edge $e=e(\gamma )\in E$ connects between two domains in *V*, if they have a common boundary component $\gamma$. The graph *G* is a subgraph of the nesting *tree*, same graph with no restriction of the domains to be contained in *B*(2*R*); though *G* may fail to be a tree, it has no cycles [12, Section 2.4]; a degree $d(\omega )$ of a vertex $\omega \in V$ in *V* precisely equals to the number of nodal components lying the boundary of $\omega$ we are to bound. We then have

$$\begin{aligned} \sum \limits _{\omega \in V}d(\omega ) = 2|E|\le 2(|V|-1) < 2 \mathcal {N}(\mathfrak {g}_{n,\alpha };2R), \end{aligned}$$

equivalently

$$\begin{aligned} \frac{1}{\mathcal {N}(\mathfrak {g}_{n,\alpha };2R)}\sum \limits _{\omega \in V}d(\omega )< 2. \end{aligned}$$

Hence, by Chebyshev’s inequality, outside $\varDelta _{6}(A)$ as in (4.13), the number of those $\omega \in V$ with $d(\omega )>L$ is at most

4.14 $$\begin{aligned} |\{\omega \in V:\, d(\omega )>L\} | \le \frac{2}{L}\cdot \mathcal {N}(g;2R) \le \frac{2}{L} \cdot AR^{n}, \end{aligned}$$

and below we will choose *L* sufficiently big so that

4.15 $$\begin{aligned} \frac{2A}{L} < \frac{\eta }{2}. \end{aligned}$$

Next we show that, with high probability, the $(n-1)$-volume of most of the components is bounded. Let $\mathcal {Z}_{\mathfrak {g}_{n,\alpha }}(2R)$ be the nodal volume of $\mathfrak {g}_{n,\alpha }$ inside *B*(2*R*)

4.16 $$\begin{aligned} \mathcal {Z}_{\mathfrak {g}_{n,\alpha }}(2R) = {\text {Vol}}_{n-1}(\mathfrak {g}_{n,\alpha }^{-1}(0) \cap B(2R)) = \sum \limits _{e(\gamma )\in E}{\text {Vol}}_{n-1}(e). \end{aligned}$$

Then, by a standard application of Lemma 2 and the stationarity of $\mathfrak {g}_{n,\alpha }$, the expectation of the nodal volume is given by

4.17 $$\begin{aligned} \mathbb {E}[\mathcal {Z}_{\mathfrak {g}_{n,\alpha }}(2R)] = c_{0}\cdot R^{n}, \end{aligned}$$

where $c_{0}=c_{0}(\mathfrak {g}_{n,\alpha }) >0$ is a positive constant, that could be evaluated explicitly in terms of *n* and $\alpha$. Hence, by (4.16) and (4.17), and Chebyshev’s inequality, outside of event of probability $\delta /2$ the number of those components with large $(n-1)$-volume is bounded:

4.18 $$\begin{aligned} \mathcal {P}(\varDelta _{7}(Q/L)):= \mathcal {P}\left\{ \left| \left\{ \gamma \in E:\, {\text {Vol}}_{n-1}(\gamma )> \frac{Q}{L}\right\} \right| > \frac{\eta }{4}\cdot R^{n} \right\} < \frac{\delta }{2}, \end{aligned}$$

provided that *Q* / *L* is sufficiently big. Since each component is lying in the boundary of at most two domains $\omega \in V$, it follows that outside of $\varDelta _{7}$ for all but at most $\frac{\eta }{2}\cdot R^{n}$ domains $\omega \in V$, *for all* components $\gamma \in E$ lying in the boundary of $\omega$ we have

4.19 $$\begin{aligned} {\text {Vol}}_{n-1}(\gamma ) \le \frac{Q}{L}. \end{aligned}$$

Now, given $\delta >0$ we choose $A>0$ sufficiently big so that (4.13) is satisfied. This forces a choice of *L* via (4.15), so that the r.h.s. of (4.14) is $<\frac{\eta }{2}\cdot R^{n}$, and then we take *Q* sufficiently big so that (4.18) is satisfied and

$$\begin{aligned} \mathcal {P}(\varDelta _{6}\cup \varDelta _{7})\le \mathcal {P}(\varDelta _{6})+\mathcal {P}(\varDelta _{7})< \delta . \end{aligned}$$

From the above, outside of $\varDelta _{6}\cup \varDelta _{7}$ for all but $\eta \cdot R^{n}$ nodal domains $\omega \in V$ we have that the number of boundary components of $\omega$ is $<L$, and the $(n-1)$-volume of *each one* of them is bounded by $\frac{Q}{L}$, and hence the $(n-1)$-volume of $\partial \omega$ is $<Q$ as claimed by this lemma. $\square$

### Proof of Proposition 1

To prove Proposition 1 we need the following lemma which is the uniform version of an obvious statement that the volume of a nodal domain depends continuously on the function as long as 0 is not a critical value.

#### Lemma 10

(cf. [13, Lemmas 6–7]) Let $R>0$ and *g* and *h* be two (deterministic) $C^2$-smooth functions on $B(2R)\subset \mathbb {R}^{n+1}$. We assume that:

1. For some $\beta >0$ we have
  $$\begin{aligned} \min \limits _{\overline{B}(2R)}\max \{ |g|,|\nabla g | \} > \beta . \end{aligned}$$
2. For some $M>0$ the $C^{2}$-norms of both *g* and *h* are bounded
  $$\begin{aligned} \Vert g \Vert _{C^{2}(B(2R))}, \Vert h \Vert _{C^{2}(B(2R))} < M. \end{aligned}$$
3. We have
  $$\begin{aligned}\Vert g-h\Vert _{C^{1}(B(R))}<b\end{aligned}$$
  for some $$b>0$$.

Then if *b* is sufficiently small (depending on $\beta$ and *M* only) there exists an injective map $\gamma \mapsto \gamma ^{h}$ between connected components $\gamma \subseteq B(R-1)$ of $g^{-1}(0)$ and connected components $\gamma ^{h}\subseteq B(R)$ of $h^{-1}(0)$ with the following properties:

1. For every $\gamma$ as above the components $\gamma$ and $\gamma ^{h}$ are “uniformly close”: there exists a smooth bijective map $\psi _{\gamma }:\gamma \rightarrow \gamma ^{h}$ so that for all $x\in \gamma$ we have
  4.20 $$\begin{aligned} \Vert \psi _{\gamma }(x)-x\Vert _{\infty } = O_{\beta ,M}(b) \end{aligned}$$
  with constants involved in the ‘*O*‘-notation depending on $\beta$ and *M* only.
2. Let $\mathcal {G}_{\gamma ,\gamma ^{h}}$ be the region enclosed between $\gamma$ and $\gamma ^{h}$. Its (signed or not) volume satisfies
  4.21 $$\begin{aligned} |{\text {Vol}}_{n}(\mathcal {G}_{\gamma ,\gamma ^{h}})|={\text {Vol}}_{n-1}(\gamma )\cdot O_{\beta ,M}(b). \end{aligned}$$

The proof of Lemma 10 is postponed until immediately after the proof of Proposition 1.

#### Proof of Proposition 1 assuming Lemma 10

We are going to show that the small exceptional event is

$$\begin{aligned} \varDelta =\bigcup \limits _{i=1}^{5}\varDelta _{i}. \end{aligned}$$

Outside $\varDelta$ we have

4.22 $$\begin{aligned}&\Vert f_{x;T}-\mathfrak {g}_{n,\alpha }\Vert _{C^{1}(\overline{B}(2R))}<b,\nonumber \\&\Vert f_{x;T}\Vert _{C^{2}(\overline{B}(2R))} , \Vert \mathfrak {g}_{n,\alpha }\Vert _{C^{2}(\overline{B}(2R))}< M, \end{aligned}$$

and assuming $b <\beta$ we also have

4.23 $$\begin{aligned} \min \limits _{\overline{B}(2R)}\max \{ |f_{x;T}|,|\nabla f_{x;T} | \}>\beta ; \; \min \limits _{\overline{B}(2R)}\max \{ |\mathfrak {g}_{n,\alpha }|,|\nabla \mathfrak {g}_{n,\alpha } | \} > \beta . \end{aligned}$$

Hence the conditions of Lemma 10 are satisfied with $h=f_{x;T}$, $g=\mathfrak {g}_{n,\alpha }$ (or the other way round) and $b,\beta$ as above.

By Lemma 10 for each nodal domain of $f_{x;T}$ lying in *B*(*R*) there is a unique nodal domain of $\mathfrak {g}_{n,\alpha }$ which is *O*(*b*) close, hence lying in $B(R+1)$. Reversing the roles of *f* and $\mathfrak {g}$ we see that the same holds for the nodal domains of $\mathfrak {g}$. We are going to prove the first inequality of (4.2), the proof of the second one is identical.

Let us consider the nodal domains of $\mathfrak {g}_{n,\alpha }$ that are inside $B(R-1)$ and of area at most $t-\xi$. Their total number is $\mathcal {N}(\mathfrak {g}_{n,\alpha },t-\xi ,R-1)$. For each of these nodal domains $\mathcal {D}$ there is a unique nodal domain $\mathcal {D}'$ of $f_{x;T}$ which is *O*(*b*) close and lies in *B*(*R*). By the estimate (4.21) of Lemma 10 we have

$$\begin{aligned} |{\text {Vol}}(\mathcal {D})-{\text {Vol}}(\mathcal {D}')|\le O_{\beta ,M}(b){\text {Vol}}_{n-1}(\partial \mathcal {D}). \end{aligned}$$

Since we excluded $\varDelta _5$, at most $\eta R^n$ of nodal domains $\mathcal {D}$ have boundary volume exceeding *Q*. For all other domains ${\text {Vol}}(\mathcal {D}')<t$ provided that *b* is so small that have $O(b)Q<\xi$. Hence, their number is bounded by $\mathcal {N}(f_{x;T},t,R)$. This means that

$$\begin{aligned} \mathcal {N}(\mathfrak {g}_{n,\alpha },t-\xi ,R-1) \le \eta R^n +\mathcal {N}(f_{x;T},t,R) \end{aligned}$$

i.e. the first inequality of (4.2).

Finally we have to show that the probability of $\varDelta$ could be made arbitrary small, using Lemmas 6–9. The argument is straightforward but the order in which we have to choose all constants is a bit fiddly.

Let $t,\xi ,\delta ,\eta >0$ be given. First we use Lemma 9 to choose *R* and *Q* sufficiently large so that $\mathcal {P}(\varDelta _5)<\delta$. From now on this value of *R* is fixed. By Lemma 8 there is $\beta >0$ such that $\mathcal {P}(\varDelta _4)<\delta$. By Lemma 7 we can choose *M* and $T_0$ large enough so that probabilities of $\varDelta _2$ and $\varDelta _3$ are bounded by $\delta$. Fix some $0<b<\beta$ sufficiently small, applying Lemma 6 and (possibly) increasing $T_0$ we make sure that $\mathcal {P}(\varDelta _1)<\delta$ as well.

All in all, for the above choice of all constants and for all $T>T_0$ we have that the probability of the exceptional event $\varDelta :=\cup _{i=1}^{5}\varDelta _{i}$ is bounded by $5\delta$. Replacing $\delta$ by $\delta /5$ we complete the proof of the proposition. $\square$

#### Proof of Lemma 10

Let $\gamma \subseteq B(R)$ be a connected component of $g^{-1}(0)$ and $x\in \gamma$ be any point on $\gamma$. By the Implicit Function Theorem we can introduce local coordinates (*z*, *y*) such that the surface near *x* can be parameterized as a graph of a smooth function *p*(*z*). In other words $\gamma$ around *x* is given by $\phi (z)=(z,p(z))$ where *p* is a smooth function on an open domain $U\subset \mathbb {R}^{n-1}$. Let

$$\begin{aligned} N(x)=N(z)=\nabla g(x)/|\nabla g(x)| \end{aligned}$$

be a unit normal vector to $\gamma$ at *x*. Since the second derivatives of *g* are uniformly bounded, there is a number $r_0=r_0(\beta ,M)>0$ depending on $\beta$ and *M* only so that

$$\begin{aligned} N(x)\cdot \nabla g(x)>\beta /2 \end{aligned}$$

on an $r_0$-neighbourhood of *x* in $\mathbb {R}^{n}$.

Now fix $x\in \gamma$ and consider

$$\begin{aligned} \zeta (r)=h(x+rN(x))=g(x+rN(x))+f(x+rN(x)), \end{aligned}$$

where $$f=h-g$$. Obviously

$$\begin{aligned} |\zeta (0)|=|h(x)|<b, \end{aligned}$$

and

$$\begin{aligned} \zeta '(r)>\beta /2-b \end{aligned}$$

for all $|r|<r_0$. For sufficiently small *b* we then have

4.24 $$\begin{aligned} \zeta '>\beta /4 \end{aligned}$$

for all $|r|<r_0$. This means that if $4b/\beta <r_0$, which is true for sufficiently small *b*, there is a unique *r* with $|r|<4b/\beta <r_0$ such that $\zeta (r)=0$. We denote it by *r*(*x*) or *r*(*z*) with $x=\phi (z)$. It is important to notice that *r* is uniformly bounded in terms of $\beta$ and *M* and for fixed $\beta$ and *M* it is *O*(*b*).

All in all, for every *b* sufficiently small (depending on $\beta$ and *M* only) the map

$$\begin{aligned} x\mapsto x+r(x)N(x) \end{aligned}$$

maps $\gamma$ onto $\gamma ^{h}$, moreover,

$$\begin{aligned} r(x)=O_{\beta ,M}(b). \end{aligned}$$

Since $\mathcal {G}_{\gamma ,\gamma ^{h}}$ is the domain between two surfaces that are $O_{\beta ,M}(b)$ close, the volume of this domain (signed or unsigned) is bounded by ${\text {Vol}}_{n-1}(\gamma )O_{\beta ,M}(b)$. This completes the proof of Lemma 10. $\square$

## Global results

### Proofs of the main results: Theorems 1,  2, 3, and 4

#### Notation 9

*(Global notation)*

1. For $$y\in \mathbb {R}$$ denote
  $$\begin{aligned}|y |_{+} = \max \{0, y\},\end{aligned}$$
  and
  $$\begin{aligned}|y |_{-} = \max \{0, -y\},\end{aligned}$$
  so that
  $$\begin{aligned}|\cdot |=|\cdot |_{+}+|\cdot |_{-}.\end{aligned}$$
2. Let $\mathcal {N}(f;t)$ be the total number of nodal domains $\omega \in \varOmega (f)$ of *f* of volume
  $$\begin{aligned}{\text {Vol}}_{n}(\omega )<t.\end{aligned}$$

#### Proposition 2

Let $\{f=f_{\alpha ,T}\}_{T>0}$ be the random fields (1.7), $c(n,\alpha )$ the Nazarov–Sodin constant of $\mathfrak {g}_{n,\alpha }$, and $t>0$ a continuity point of

$$\begin{aligned} \varPsi (\cdot )=\varPsi _{n,\alpha }(\cdot ), \end{aligned}$$

as in the formulation of Theorem 8. Then the following holds:

5.1 $$\begin{aligned} \mathbb {E}\left[ \left| \frac{\mathcal {N}(f,t/T^n)}{c(n,\alpha ){\text {Vol}}_{n}(\mathcal {M})\cdot T^{n}} - \varPsi (t) \right| _{\pm } \right] \rightarrow 0, \end{aligned}$$

that is, (5.1) is claimed for both $|\cdot |_{+}$ and $|\cdot |_{-}$, as in Notation 5.1.

Proposition 2 will be proved in Sect. 5.2.2 for $|\cdot |_{+}$ only, with the proof for $|\cdot |_{-}$ following along similar, but easier, lines, with no need to excise the very small and very long domains (see Sect. 5.2.1).

#### Proof of Theorem 2 assuming Proposition 2

Combining both estimates (5.1) of Proposition 2 (i.e, $|\cdot |_{+}$ and $|\cdot |_{-}$) implies that for every $t>0$ continuity point of $\varPsi (\cdot )$ we have

5.2 $$\begin{aligned} \mathbb {E}\left[ \left| \frac{\mathcal {N}(f,t/T^n)}{c(n,\alpha ){\text {Vol}}(\mathcal {M})\cdot T^{n}} - \varPsi (t) \right| \right] \rightarrow 0, \end{aligned}$$

which is not quite the same as the statement (1.9) of Theorem 2 as the denominator needs to be replaced by $\mathcal {N}(f)$ rather than

$$\begin{aligned}c(n,\alpha ){\text {Vol}}(\mathcal {M})\cdot T^{n}.\end{aligned}$$

To this end we use the triangle inequality to write

5.3 $$\begin{aligned} \mathbb {E}\left[ \left| \frac{\mathcal {N}(f,t/T^n)}{\mathcal {N}(f)} - \varPsi (t) \right| \right]&\le \mathbb {E}\left[ \left| \frac{\mathcal {N}(f,t/T^n)}{\mathcal {N}(f)} - \frac{\mathcal {N}(f,t/T^n)}{c(n,\alpha ){\text {Vol}}(\mathcal {M})\cdot T^{n}} \right| \right] \nonumber \\&+ \mathbb {E}\left[ \left| \frac{\mathcal {N}(f,t/T^n)}{c(n,\alpha ){\text {Vol}}(\mathcal {M})\cdot T^{n}} - \varPsi (t) \right| \right] . \end{aligned}$$

Since the second summand of the r.h.s. of (5.3) vanishes by (5.2), we only need to take care of the first one. We have

5.4 $$\begin{aligned}&\mathbb {E}\left[ \left| \frac{\mathcal {N}(f,t/T^n)}{\mathcal {N}(f)} - \frac{\mathcal {N}(f,t/T^n)}{c(n,\alpha ){\text {Vol}}(\mathcal {M})\cdot T^{n}} \right| \right] \nonumber \\&= \mathbb {E}\left[ \frac{\mathcal {N}(f,t/T^n)}{c(n,\alpha ){\text {Vol}}(\mathcal {M})\cdot \mathcal {N}(f)} \cdot \left| \frac{\mathcal {N}(f)}{T^{n}} - c(n,\alpha ){\text {Vol}}(\mathcal {M}) \right| \right] \rightarrow 0, \end{aligned}$$

by (1.8), as it is obvious that

$$\begin{aligned}\frac{\mathcal {N}_{\varOmega }(f,t/T^n)}{\mathcal {N}(f)} \le 1.\end{aligned}$$

We finally substitute (5.2) and (5.4) into (5.3) to yield the statement (1.9) of Theorem 2. $\square$

#### Proof of Theorem 1

The first assertion of Theorem 1 is a particular case of the statement of Theorem 2 with $\mathcal {M}=\mathcal {S}^{2}$ the round 2-sphere, and $\alpha =1$. The second assertion of Theorem 1 is the content of Theorem 4 (which, in this case, follows directly from Theorem 7). $\square$

#### Proofs of Theorems 3 and 4

By Theorem 2 the distribution function is universal and from (2.2) we know the spectral measures. In the case $\alpha <1$ it satisfies the axioms $(\rho 1)-(\rho 3)$ and $(\rho 4^{*})$ and Theorem 3 follows directly from Theorem 6. In the case $\alpha =1$ the limiting field is the random plane wave and Theorem 4 follows from Theorem 7. $\square$

### Proof of Proposition 2

#### Excising the very small and very long domains

##### Definition 3

Let $\xi , D>0$ be parameters.

1. A domain $\omega \in \varOmega (f)$ of $f=f_{\alpha ;T}$ is called ‘$\xi$-*small*’ if its *n*-dimensional Riemannian volume in $\mathcal {M}$ is
  $$\begin{aligned}{\text {Vol}}_{\mathcal {M}}(\omega )<\xi T^{-n}.\end{aligned}$$
  Let $\mathcal {N}_{\xi -sm}(f)$ be the total number of $\xi$-small domains (components) of *f* in $\mathcal {M}$.
2. For $D>0$, a nodal domain $\omega \in \varOmega (f)$ is *D*-*long* if its diameter is $>D/T$. Let $\mathcal {N}_{D-long}(f)$ be the total number of the *D*-long domains of *f*.
3. Given parameters $D,\xi > 0$ a nodal domain $\omega \in \varOmega (f)$ is ($D,\xi$)-*normal* (or simply normal), if it is not $\xi$-small nor *D*-long. For $t>0$ let $\mathcal {N}_{norm}(f,t)$ be the total number of $(\xi ,D)$-normal domains of *f* of volume $<t,$ and for $x\in \mathcal {M}$, $r>0$ let $\mathcal {N}_{norm}(f_{L},t;x,r)$ (resp. $\mathcal {N}_{norm}^{*}(f_{L},t;x,r)$) be the number of those contained in $B_{x}(r)$ (resp. intersecting $\overline{{B}_{x}}(r)$). Finally, if *t* is omitted, then it is assumed to be infinite $t=\infty$, i.e. no restriction on the domain volume is imposed.

By the definition of normal domains, for every $t\in \mathbb {R}\cup \{\infty \}$ we have

5.5 $$\begin{aligned} \mathcal {N}_{norm}^{*}(f,t;x,r) \le \mathcal {N}_{norm}\left( f,t;x,r+\frac{D}{T}\right) \end{aligned}$$

(as we discarded the very long ovals), and

5.6 $$\begin{aligned} \mathcal {N}_{norm}(f;x,r) \le \xi ^{-1}T^{n} {\text {Vol}}_{\mathcal {M}}(B_{x}(r)), \end{aligned}$$

by the natural volume estimate.

##### Lemma 11

(cf. [13, Lemma 9], see also [12] for a more detailed proof) There exist constants $c,C>0$ so that we have the following estimate on the number of $\xi$-small components:

$$\begin{aligned} \limsup \limits _{T\rightarrow \infty }\frac{\mathbb {E}[\mathcal {N}_{\xi -sm}(f_{\alpha ;T})]}{T^{n}} \le C\cdot \xi ^{c}. \end{aligned}$$

##### Lemma 12

(cf. [13, Lemma 8]) There exists a constant $C>0$ such that the following bound holds for the number of *D*-long components:

$$\begin{aligned} \limsup \limits _{T\rightarrow \infty }\frac{\mathbb {E}[\mathcal {N}_{D-long}(f_{\alpha ;T})]}{T^{n}} \le C\cdot \frac{1}{D}. \end{aligned}$$

#### Proof of Proposition 2

##### Proposition 3

Let $\xi , D>0$ be fixed, and

$$\begin{aligned} \varPsi =\varPsi _{n,\alpha }. \end{aligned}$$

Then for every point of continuity $t>0$ of $\varPsi (\cdot )$ the following holds:

5.7 $$\begin{aligned} \mathbb {E}\left[ \left| \frac{\mathcal {N}_{norm}(f_{\alpha ;T},t/T^{n})}{c(n,\alpha ){\text {Vol}}(\mathcal {M})\cdot T^{n}} - \varPsi (t) \right| _{+} \right] \rightarrow 0. \end{aligned}$$

The proof of Proposition 3 will be given in Sect. 5.3.

##### Proof of Proposition 2 assuming Proposition 3

The estimate (5.1) for $|\cdot |_{+}$ follows directly from (5.7), lemmas 12–11, and the triangle inequality for $|\cdot |_{+}$. The proof of (5.1) for $|\cdot |_{-}$ follows along the same lines, except that in this case we do not need to excise the very small and very long domains (which makes the proof somewhat simpler). $\square$

### Proof of Proposition 3

We need to formulate a couple of auxiliary lemmas.

#### Lemma 13

(cf. [13] Lemma 1, and Lemma 5 in the scale-invariant case) Given $\epsilon > 0$, there exists $\eta >0$ such that for every $r<\eta$, $t>0$

5.8 $$\begin{aligned} \begin{aligned}&(1-\epsilon )\int \limits _{\mathcal {M}}\frac{\mathcal {N}_{norm}(f_{\alpha ;T},t;x,r)}{{\text {Vol}}(B(r))}dx \le \mathcal {N}_{norm}(f_{\alpha ;T},t) \\&\le (1+\epsilon )\int \limits _{\mathcal {M}}\frac{\mathcal {N}_{norm}^{*}(f_{\alpha ;T},t;x,r)}{{\text {Vol}}(B(r))}dx \end{aligned} \end{aligned}$$

The proof of Lemma 13 is very similar to the one of Lemma 5 and is omitted here.

#### Proof of Proposition 3

For convenience in course of this proof we will assume that $\mathcal {M}$ is unit volume

5.9 $$\begin{aligned} {\text {Vol}}(\mathcal {M})=1, \end{aligned}$$

and recall that $f=f_{\alpha ;T}$ are the band-limited random fields (1.7), and that we use the shorthand

$$\begin{aligned}\varPsi (t)=\varPsi _{n,\alpha }(t)\end{aligned}$$

as in the formulation of Proposition 3. Let $\epsilon >0$ be a small number. To bound the l.h.s. of (5.7) we let $R>0$ be sufficiently big, so that both $R/T<\eta$ as in Lemma 13, and *D* / *R* is sufficiently small, so that the following holds

5.10 $$\begin{aligned} \left| \frac{{\text {Vol}}_{\mathcal {M}}(B_{x}(R/T))}{{\text {Vol}}(B(R/T))} -1\right|< & {} \epsilon ,\nonumber \\ \frac{{\text {Vol}}(B(R+D))}{{\text {Vol}}(B(R))}< & {} 1+\epsilon \end{aligned}$$

uniformly for $$x\in \mathcal {M}$$.

Now apply Lemma 13 with $r=\frac{R}{ T}$ and *t* replaced by $\frac{t}{T^{n}}$; by the triangle inequality for $|\cdot |_{+}$ we have

5.11 $$\begin{aligned}&\mathbb {E}\left[ \left| \frac{\mathcal {N}_{norm}(f_{\alpha ;T},t/T^{n})}{c(n,\alpha )\cdot T^{n}} - \varPsi (t) \right| _{+} \right] \nonumber \\&\le \mathbb {E}\left[ \int \limits _{\mathcal {M}}\left| (1+2\epsilon )\frac{\mathcal {N}_{norm}^{*}(f_{\alpha ;T},t/T^{n};x,R/T)}{c(n,\alpha )\cdot {\text {Vol}}B(R+D)} -\varPsi (t)\right| _{+}dx \right] \nonumber \\&\le \mathbb {E}\left[ \int \limits _{\mathcal {M}}\left| \frac{\mathcal {N}_{norm}(f_{\alpha ;T},t/T^{n};x,(R+D)/T)}{c(n,\alpha )\cdot {\text {Vol}}B(R+D)}-\varPsi (t)\right| _{+}dx \right] \nonumber \\&\quad +O\left( \epsilon \cdot \int \limits _{\mathcal {M}}\frac{\mathbb {E}[\mathcal {N}_{norm}(f_{\alpha ;T};x,(R+D)/T)]}{{\text {Vol}}B(R+D)}dx \right) , \end{aligned}$$

by (5.5) and (5.10). Observe that the integrand

$$\begin{aligned}\frac{\mathbb {E}[\mathcal {N}_{norm}(f_{\alpha ;T};x,(R+D)/T)]}{{\text {Vol}}B(R+D)}\end{aligned}$$

is uniformly bounded by Lemma 4. Hence (5.11) is

$$\begin{aligned} \begin{aligned}&\mathbb {E}\left[ \left| \frac{\mathcal {N}_{norm}(f_{\alpha ;T},t/T^{n})}{c(n,\alpha )\cdot T^{n}} - \varPsi (t) \right| _{+} \right] \\&\le \mathbb {E}\left[ \int \limits _{\mathcal {M}}\left| \frac{\mathcal {N}_{norm}(f_{\alpha ;T},t/T^{n};x,(R+D)/T)}{c(n,\alpha )\cdot {\text {Vol}}B(R+D)}-\varPsi (t)\right| _{+}dx\right] + O(\epsilon ). \end{aligned} \end{aligned}$$

It is then sufficient to prove that

5.12 $$\begin{aligned}&\mathbb {E}\left[ \int \limits _{\mathcal {M}} \left| \frac{\mathcal {N}_{norm}(f_{\alpha ;T},t/T^{n};x,(R+D)/T)}{c(n,\alpha )\cdot {\text {Vol}}B(R+D)}-\varPsi (t)\right| _{+}dx\right] \nonumber \\&\quad = \int \limits _{\varDelta }\int \limits _{\mathcal {M}} \left| \frac{\mathcal {N}_{norm}(f_{\alpha ;T},t/T^{n};x,(R+D)/T)}{c(n,\alpha )\cdot {\text {Vol}}B(R+D)}-\varPsi (t)\right| _{+}dx d\mathcal {P}(\omega ) \rightarrow 0, \end{aligned}$$

where $\varDelta$ is the underlying probability space, and $\mathcal {P}$ is the probability measure on $\varDelta$. Now consider the event

$$\begin{aligned} \varDelta _{T,t;x,R} = \left\{ \left| \frac{\mathcal {N}(f_{\alpha ;T},t/T^{n};x,R/T)}{c(n,\alpha )\cdot {\text {Vol}}B(R+D)}-\varPsi (t)\right| >\epsilon \right\} . \end{aligned}$$

Then, recalling the assumption of Proposition 3 on *t* (i.e. that $\varPsi (\cdot )$ is continuous at *t*), Theorem 8 implies that for all $x\in \mathcal {M}$

5.13 $$\begin{aligned} \lim \limits _{R\rightarrow \infty }\limsup \limits _{T\rightarrow \infty } \mathcal {P}(\varDelta _{T,t;x,R} ) =0. \end{aligned}$$

We claim that the above implies that there exists a sequence $\{R_{j}\}_{j\rightarrow \infty }$ of values $R=R_{j}\rightarrow \infty$ so that the limit (5.13) is almost uniform w.r.t. $x\in X$, that is, for every $\rho >0$ there exists $\mathcal {M}_{\rho } \subseteq \mathcal {M}$ of volume

$$\begin{aligned}{\text {Vol}}\mathcal {M}_{\rho }>1-\rho ,\end{aligned}$$

such that

5.14 $$\begin{aligned} \varliminf \limits _{R\rightarrow \infty }\varlimsup \limits _{T\rightarrow \infty } \sup \limits _{x\in \mathcal {M}_{\rho }}\mathcal {P}(\varDelta _{T,t;x,R+D}) =0. \end{aligned}$$

To see (5.14) we first apply an Egorov-type theorem on the limit in (5.13) w.r.t. $R\rightarrow \infty$: working with the sets

$$\begin{aligned}E_{n,k} = \bigcup \limits _{R>n \text { integer}}\left\{ x\in \mathcal {M}:\, \mathcal {P}(\varDelta _{T,t;x,R+D}) > \frac{1}{k} \text { for } T=T_{j}\rightarrow \infty \right\} \end{aligned}$$

yields that for some $M_{\rho }$ with

$$\begin{aligned} {\text {Vol}}(M_{\rho })>1-\frac{\rho }{2} \end{aligned}$$

we have

$$\begin{aligned} \lim \limits _{R\rightarrow \infty }\sup \limits _{x\in \mathcal {M}_{\rho }}\varlimsup \limits _{T\rightarrow \infty } \mathcal {P}(\varDelta _{T,t;x,R+D}) =0; \end{aligned}$$

this is not quite the same as the claimed result (5.14), as the order of $\sup \nolimits _{x\in \mathcal {M}_{\rho }}$ and the $\limsup$ w.r.t. $T\rightarrow \infty$ is wrong. We use an Egorov-type argument once again, w.r.t. the limit $\varlimsup \nolimits _{T\rightarrow \infty }$ to mollify this. Fix an integer $r>0$, and let $R=R(r)>0$ sufficiently big so that

5.15 $$\begin{aligned} \sup \limits _{x\in \mathcal {M}_{\rho }}\varlimsup \limits _{T\rightarrow \infty } \mathcal {P}(\varDelta _{T,t;x,R+D})<\frac{1}{r}. \end{aligned}$$

Define the monotone decreasing sequence of sets

$$\begin{aligned} F_{m} = \bigcup \limits _{T>m}\left\{ x\in \mathcal {M}_{\rho }:\, \mathcal {P}(\varDelta _{T,t;x,R+D}) > \frac{2}{r} \right\} . \end{aligned}$$

Since, by (5.15),

$$\begin{aligned} \bigcap \limits _{m\ge 1} F_{m}=\emptyset , \end{aligned}$$

we may find $m=m(r)$ sufficiently big so that ${\text {Vol}}(F_{m(r)})<\frac{\rho }{2^{r+1}}$. Therefore the claimed result (5.14) holds on

$$\begin{aligned} M_{\rho }\setminus \bigcup \limits _{r\ge 1}F_{m(r)}, \end{aligned}$$

i.e. further excising the set

$$\begin{aligned} \bigcup \limits _{r\ge 1}F_{m(r)} \end{aligned}$$

of volume $<\frac{\rho }{2}$ from $\mathcal {M}_{\rho }$.

We then write the integral (5.12) as

5.16 $$\begin{aligned}&\int \limits _{\varDelta }\int \limits _{\mathcal {M}} \left| \frac{\mathcal {N}_{norm}(f_{\alpha ;T},t/T^{n};x,(R+D)/T)}{c(n,\alpha )\cdot {\text {Vol}}B((R+D))}-\varPsi (t)\right| _{+}dx d\mathcal {P}(\omega )\nonumber \\&\quad =\int \limits _{\mathcal {M}}\int \limits _{\varDelta _{T,t;x,R+D}} +\int \limits _{\mathcal {M}}\int \limits _{\varDelta \setminus \varDelta _{T,t;x,R+D}}. \end{aligned}$$

First, on $\varDelta {\setminus }\varDelta _{T,t;x,R+D}$, the integrand of (5.16) is

$$\begin{aligned} \begin{aligned}&\left| \frac{\mathcal {N}_{norm}(f_{\alpha ;T},t/T^{n};x,(R+D)/T)}{c(n,\alpha )\cdot {\text {Vol}}B((R+D)/T)}-\varPsi (t)\right| _{+} \\&\le \left| \frac{\mathcal {N}(f_{\alpha ;T},t/T^{n};x,(R+D)/T)}{c(n,\alpha )\cdot {\text {Vol}}B((R+D)/T)}-\varPsi (t)\right| \le \epsilon , \end{aligned} \end{aligned}$$

and hence the contribution of this range is

5.17 $$\begin{aligned}&\int \limits _{\mathcal {M}}\int \limits _{\varDelta \setminus \varDelta _{T,t;x,R+D}} \left| \frac{\mathcal {N}_{norm}(f_{\alpha ;T},t/T^{n};x,(R+D)/T)}{c(n,\alpha )\cdot {\text {Vol}}B((R+D)/T)}-\varPsi (t)\right| _{+} d\mathcal {P}(\omega )dx\nonumber \\&\quad \le \int \limits _{\mathcal {M}}\int \limits _{\varDelta \setminus \varDelta _{T,t;x,R+D}}\epsilon d\mathcal {P}(\omega )dx \le \epsilon . \end{aligned}$$

On $\varDelta _{T,t;x,R+D}$ we use the volume estimate (5.6) yielding uniformly on $x\in \mathcal {M}$

5.18 $$\begin{aligned}&\int \limits _{\varDelta _{T,t;x,R+D}} \left| \frac{\mathcal {N}_{norm}(f_{\alpha ;T},t/T^{n};x,(R+D)/T)}{c(n,\alpha )\cdot {\text {Vol}}B(R+D)}-\varPsi (t)\right| _{+} d\mathcal {P}(\omega )\nonumber \\&\quad \le \int \limits _{\varDelta _{T,t;x,R+D}} \left| \frac{\mathcal {N}_{norm}(f_{\alpha ;T},t/T^{n};x,(R+D)/T)}{c(n,\alpha )\cdot {\text {Vol}}B(R+D)}\right| _{+}d\mathcal {P}(\omega )\nonumber \\&\quad \le \int \limits _{\varDelta _{T,t;x,R+D}}\xi ^{-1}T^{n}\frac{{\text {Vol}}_{\mathcal {M}}(B_{x}((R+D)/T))}{c(n,\alpha )\cdot {\text {Vol}}B(R+D)}d\mathcal {P}(\omega ) \nonumber \\&\quad \le (1+\epsilon )\xi ^{-1}\mathcal {P}(\varDelta _{T,t;x,R+D}) \end{aligned}$$

(where, by the obvious properties of $|\cdot |_{+}$, to obtain the first inequality we omitted the non-negative $\varPsi (t)$). Similarly to the above, uniformly for $\omega \in \varDelta$

5.19 $$\begin{aligned} \int \limits _{\mathcal {M}\setminus \mathcal {M}_{\rho }} \left| \frac{\mathcal {N}_{norm}(f_{\alpha ;T},t/T^{n};x,(R+D)/T)}{c(n,\alpha )\cdot {\text {Vol}}B(R+D)}-\varPsi (t)\right| _{+} dx \le (1+\epsilon )\xi ^{-1}\rho . \end{aligned}$$

The uniform estimates (5.18) and (5.19) imply that

$$\begin{aligned} \begin{aligned}&\int \limits _{\mathcal {M}}\int \limits _{\varDelta _{L,t;x,R+D}} \left| \frac{\mathcal {N}_{norm}(f_{\alpha ;T},t/T^{n};x,(R+D)/T)}{c(n,\alpha )\cdot {\text {Vol}}B(R+D)}-\varPsi (t)\right| _{+} \mathcal {P}(\omega )dx \\&\le (1+\epsilon )\xi ^{-1} (\sup \limits _{x\in \mathcal {M}_{\rho }}\mathcal {P}(\varDelta _{T,t;x,R+D}) + \rho ). \end{aligned} \end{aligned}$$

Upon substituting the latter estimate and (5.17) into (5.16), and then to the integral (5.12), we finally obtain

$$\begin{aligned} \begin{aligned}&\mathbb {E}\left| \int \limits _{\mathcal {M}} \frac{\mathcal {N}_{norm}(f_{\alpha ;T},t/T^{n};x,(R+D)/T)}{c(n,\alpha )\cdot {\text {Vol}}B(R+D)}-\varPsi (t)\right| _{+}dx\\&\le \epsilon + (1+\epsilon )\xi ^{-1} (\sup \limits _{x\in \mathcal {M}_{\rho }}\mathcal {P}(\varDelta _{T,t;x,R+D}) + \rho ), \end{aligned} \end{aligned}$$

which could be made arbitrarily small for each sufficiently small choice of $\xi$ excising the very small components, and using (5.14). This concludes the proof of (5.12), sufficient to yield the conclusion of Proposition 3. $\square$

## Final remark: volumes of the nodal components

### Volume distribution of boundary components

We would like to point out that the methods we are using are rather general and with minimal change one may prove other results. These in particular include similar results about the $(n-1)$ dimensional volumes of nodal components or boundaries of nodal domains instead of the *n* dimensional volume of the nodal domains. The analogue of Theorem 5 is proved in exactly the same way using Kac–Rice for the bound and ergodic theory for the existence of the limit. Going from the planar case to the Riemannian one is also straighforward. All these results could be rewritten line-by-line, with the definition of $\mathcal {N}$ essentially the only change.

The only difference is in the results stating that the limit functions are strictly increasing i.e. Theorems 6 and 7. The main idea is still the same: we have to create a deterministic example with a nodal component of (approximately) given size, and then show that small perturbations do not significantly alter its size with positive probability. For $\alpha <1$ the argument is essentially the same as for the volume of nodal domains. In this case the corresponding Hilbert space $\mathcal H$ is dense in any $C^k(Q)$, hence we can construct any non-degenerate example. For $\alpha =1$, the same family of domains works for the nodal sets, the minimal volume will be the volume of the surface volume of the sphere of radius $j_{n/2-1,1}$ instead of the the *n* dimensional volume of the corresponding ball. (This follows from the isoperimetric inequality.) The only real difference is in Lemma 10: controlling the $C^1$ norm is not sufficient in order to control the change of the boundary volume. But it is not very difficult to show that if the perturbation has a small $C^2$-norm *b*, then the ratio of boundary volumes of the original and perturbed domains is $1+O(b)$ where the constant in *O*(*b*) depends only on the $C^2$ norms.

### Coupling between domain boundary volumes

Finally, we point that it is possible to consider the *joint* distribution of

$$\begin{aligned} ({\text {Vol}}_n(\omega ),{\text {Vol}}_{n-1}(\partial \omega )) \end{aligned}$$

where $\omega$ is a nodal domain. The existence of the scaling limit is established via an identical argument to the above. Constructing a deterministic example in the case $\alpha <1$ also follows along the lines of the argument in this manuscript. This would give that the limiting distribution $\varPsi (t,l)$ is strictly increasing in both *t* and *l* for all $t>0$ and $l>l_0(t)$, where $l_0(t)$ is the surface area of a sphere with volume *t* (follows from the isoperimetric inequality).

**Fig. 1:**
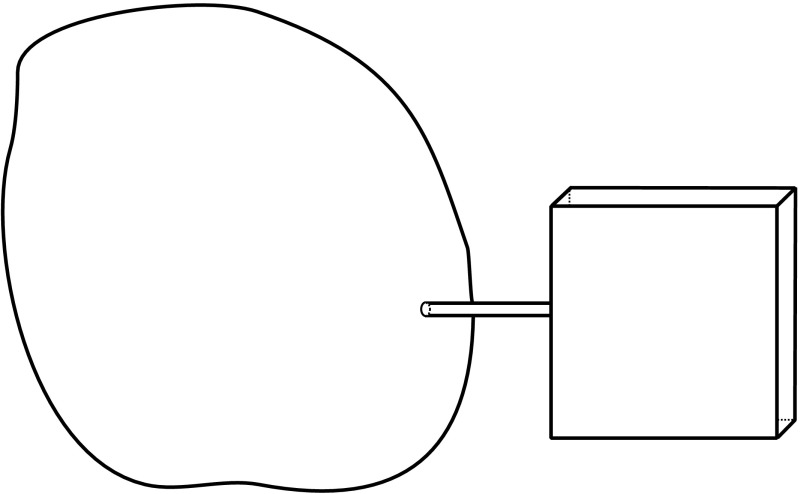
Connecting a thin plate by a thin tube

The case $\alpha =1$ is more complicated. From the isoperimetric inequality, for each $t>t_0$, the size of the boundary must be $\ge l_0(t)$. But it is clear that this is not the best possible lower bound, as otherwise the domain would be a ball and its main eigenvalue would be larger than 1 unless $t=t_0$. On the other hand, there *exists* a deterministic infimum of the boundary volume over all domains that have volume $t\ge t_0$ and the main eigenvalue 1. We denote this infimum by $l_1(t)$. There exists a domain for which the boundary is *arbitrary close* to $l_1(t)$. Next we attach to this domain a very thin tube and at the end of this tube we attach a very thin plate, see Fig. 1. This only slightly perturbs the volume, does not alter the main eigenvalue significantly, but increases the boundary volume a lot. After that we take a small perturbation to make the domain real analytic and use Lax-Malgrange to approximate the main eigenfunction by a plane wave. This way we can construct a nodal domain with eigenvalue 1, volume arbitrary close to $t\ge t_0$ and boundary volume arbitrary close to $l\ge l_1(t)$.

## Footnotes

1 Note the different normalization as compared to [12].

2 To make sense of the distribution of $\mathcal {N}(F,\cdot ;\cdot )$ it is essential to show that the latter is a *random variable*, i.e. a measurable function on the sample space. Fortunately, the proof of a similar statement, given in [12, Appendix A], is sufficiently robust to cover our case and all the other similar quantities of this manuscript; from this point on we will neglect any issue of measurability.
